# Beyond GLP-1: efficacy and safety of dual and triple incretin agonists in personalized type 2 diabetes care—a systematic review and network meta-analysis

**DOI:** 10.1007/s00592-025-02534-y

**Published:** 2025-06-05

**Authors:** Kangling Yan, Haichuan Yu, Benoît Blaise

**Affiliations:** 1 School of Medical Sciences - Faculty of Medicine and Health, University of Sydney, City Road, Sydney, NSW 2006 Australia; 2 Wuhan University, Wuhan City, 430061 Hubei China; 3 Faculty of Pharmacy, Université Paris-Saclay, 92290 Châtenay-Malabry, Paris France

**Keywords:** Type 2 Diabetes Mellitus, Incretin-Based Agonists, Dual and Triple Agonists, Glycemic Control, Network Meta-Analysis, Adverse Events

## Abstract

**Background:**

Dual and triple incretin-based agonists, targeting combinations of GLP-1, GIP, and glucagon receptors, represent an innovative approach in T2DM care. However, comparative efficacy and safety analyses tailored to receptor-specific strategies are limited.

**Purpose:**

This systematic review and network meta-analysis uniquely evaluates the efficacy and safety of dual and triple incretin agonists compared to standard therapies, offering insights into personalized, receptor-specific T2DM therapies.

**Data Sources:**

Systematic searches in PubMed, Web of Science, Cochrane Library, and Embase (up to July 2024) identified RCTs.

**Study Selection:**

Trials assessing dual or triple incretin therapies in T2DM with outcomes on weight, HbA1c, FBG, AEs, and SAEs were included.

**Data Extraction:**

Data on efficacy and safety were extracted by independent reviewers and assessed for quality using the NIH Quality Assessment Tool.

**Data Synthesis:**

Retatrutide achieved the greatest weight reduction (MD: − 8.601; 95% CrI: − 11.20 to − 5.95) while Tirzepatide was most effective in lowering FBG (MD: − 57.30) and HbA1c ( − 1.88), with 95% CrIs of − 65.41 to − 48.9 and − 2.15 to − 1.64 respectively. Tirzepatide (RR 1.15) and Cotadutide (1.38) increased AEs, while Semaglutide reduced SAEs (0.35); 95% Crls: 1.04–1.33, 1.16–1.68, and 0.13–0.78, respectively.

**Limitations:**

Small sample sizes, short study durations, and reliance on indirect comparisons in some cases may limit the certainty of these findings. Direct head-to-head trials are needed to confirm these results.

**Conclusion:**

Receptor-specific targeting optimizes T2DM treatment, with Semaglutide supporting glycemic control, Tirzepatide enhancing weight loss and glucose regulation, and Retatrutide potentially offering broader metabolic benefits, advancing receptor-targeted, personalized therapy.

**PROSPERO registration number:**

CRD42024532368

**Supplementary Information:**

The online version contains supplementary material available at 10.1007/s00592-025-02534-y.

## Introduction

Type 2 diabetes mellitus (T2DM) is a prevalent metabolic disease with serious complications, posing a major global health challenge. In 2019, the International Diabetes Federation (IDF) reported that approximately 463 million individuals worldwide have diabetes, and this number is expected to rise to 700 million by 2045 [1]. T2DM is primarily characterized by chronic hyperglycemia due to insulin resistance and progressive pancreatic β-cell dysfunction. These mechanisms are closely tied to rising obesity rates, which further exacerbate the condition [1]. Rising obesity rates are closely linked to these mechanisms, further exacerbating insulin resistance and glucose metabolism disruptions, thereby accelerating disease progression [2].

Given the strong association between obesity and T2DM, effective weight management is integral to diabetes care, as weight loss improves insulin sensitivity, glycemic control, and reduces cardiovascular complications [2]. However, many patients struggle to achieve sustained weight loss through lifestyle changes alone, emphasizing the need for pharmacologic therapies targeting both glycemic control and weight reduction [3]. Glucagon-like peptide-1 (GLP-1) receptor agonists marked a key advancement in T2DM treatment by enhancing insulin secretion, inhibiting glucagon release, slowing gastric emptying, and reducing appetite, thus supporting both glycemic control and weight loss [4, 5]. Semaglutide, for example, has demonstrated substantial efficacy in reducing both HbA1c and body weight [3, 6].

Building on the success of GLP-1 receptor agonists, dual and triple agonists that activate additional receptors, such as glucose-dependent insulinotropic polypeptide (GIP) and glucagon receptors (GCGR), have been developed. These multi-incretin receptor therapies, like Tirzepatide (a dual GLP-1/GIP agonist) and Retatrutide (a triple GLP-1/GIP/GCGR agonist), have shown superior efficacy over single-receptor agonists in weight reduction and blood glucose control [5, 7]. Despite their promise, most previous studies have focused on GLP-1 receptor agonists alone or evaluated polyagonists in non-diabetic populations, mainly for weight management [8, 9]. Even recent meta-analyses, such as Yao et al. (2024), review GLP-1 receptor agonists for T2DM but lack receptor-specific comparisons that are essential for tailoring treatments to individual patient needs [10].

To address this gap, this study conducted a comprehensive network meta-analysis to evaluate a broader range of incretin-based multi-receptor therapies, incorporating additional agents not included in recent studies (e.g. Cotadutide, SAR425899, RG7697).

Since multiple dual and triple incretin-based agonists have now been developed, but direct head-to-head comparisons among them remain scarce, network meta-analysis (NMA) allows for the integration of both direct and indirect evidence, providing a comprehensive assessment of the relative efficacy and safety of these therapies. By synthesizing available data, this approach offers valuable insights into the comparative performance of different co-agonists, ultimately guiding future research directions and informing clinical decision-making.

By examining GLP-1/GIP agonists, GLP-1/GCGR agonists, and GLP-1/GIP/GCGR agonists alongside standard comparators like insulins, this study provides a detailed assessment of their effects on glycemic control, weight loss, and safety. This receptor-specific approach offers nuanced insights into how various receptor combinations and affinities influence clinical outcomes, supporting more personalized, evidence-based diabetes care.

## Methods

This systematic review followed the Preferred Reporting Items for Systematic Reviews and Meta-Analyses (PRISMA) guideline and the PRISMA Extension for Network Meta-analyses, as detailed in *The PRISMA Extension Statement for Reporting of Systematic Reviews Incorporating Network Meta-analyses* [11]. It was registered in PROSPERO (registration No.: CRD42024532368).

### Literature search and data source

A comprehensive literature search in PubMed, Web of Science, Cochrane Library, and Embase using keywords such as “T2DM”, “coagonist”, “Retatrutide” and “Cotadutide” along with related terms, to capture all relevant studies up to July 2024. No language restrictions were applied. Full search details are in Supplementary Material.

### Study selection

Two researchers independently screened studies using established criteria, with a third researcher reviewing the results. This study included randomized controlled trials (RCTs) with T2DM patients treated with dual or triple incretin-based therapies targeting GLP-1, GIP and glucagon receptors, compared to other active T2DM treatments. Primary outcome measures included weight change, HbA1c levels and fasting blood glucose (FBG), with adverse events (AEs) and serious adverse events (SAEs) as secondary outcomes. Non-original research (conference abstracts, editorials, reviews) was excluded.

### Assessment of bias risk and data extraction

Two researchers independently assessed bias risk and extracted data using a pre-designed form, with a third researcher verifying for accuracy. Study quality was evaluated with the NIH Quality Assessment Tool for Controlled Intervention Studies (NIH-QAT), covering 14 criteria such as randomization, blinding, statistical methods, and overall risk of bias. Studies were rated as good, average, or poor. Extracted data included study details, patient demographics, intervention specifics, comparator information, and outcomes.

### Statistical analysis

A Bayesian network meta-analysis was conducted using R (version 4.4.0) with the “gemtc” package (1.0–2) to evaluate efficacy and safety differences between dual and triple agonists in T2DM treatment. This framework allowed for complex direct and indirect comparisons, with random-effects models enhancing result accuracy. Model convergence quality was assessed via the potential scale reduction factor (PSRF), aiming for values near 1. Treatment rankings were determined using the surface under the cumulative ranking curve (SUCRA), league tables, and forest plots, with mean differences (MDs) and 95% credible intervals (95% CrI) for effect size. Results were considered significant if the 95% CrI excluded 0. Inconsistency tests (using “mtc.nodesplit”) assessed the alignment of direct and indirect evidence, and heterogeneity was evaluated with the “mtc.anohe” function to ensure study result homogeneity.

To further explore the impact of dosage and treatment duration on effect sizes, we conducted an additional stratified network meta-analysis, categorizing treatments based on drug type, dose, and duration of administration. This analysis allowed us to assess whether higher doses or longer treatment durations influenced the efficacy of different therapies.

## Results

### Literature screening

**Fig. 1:**
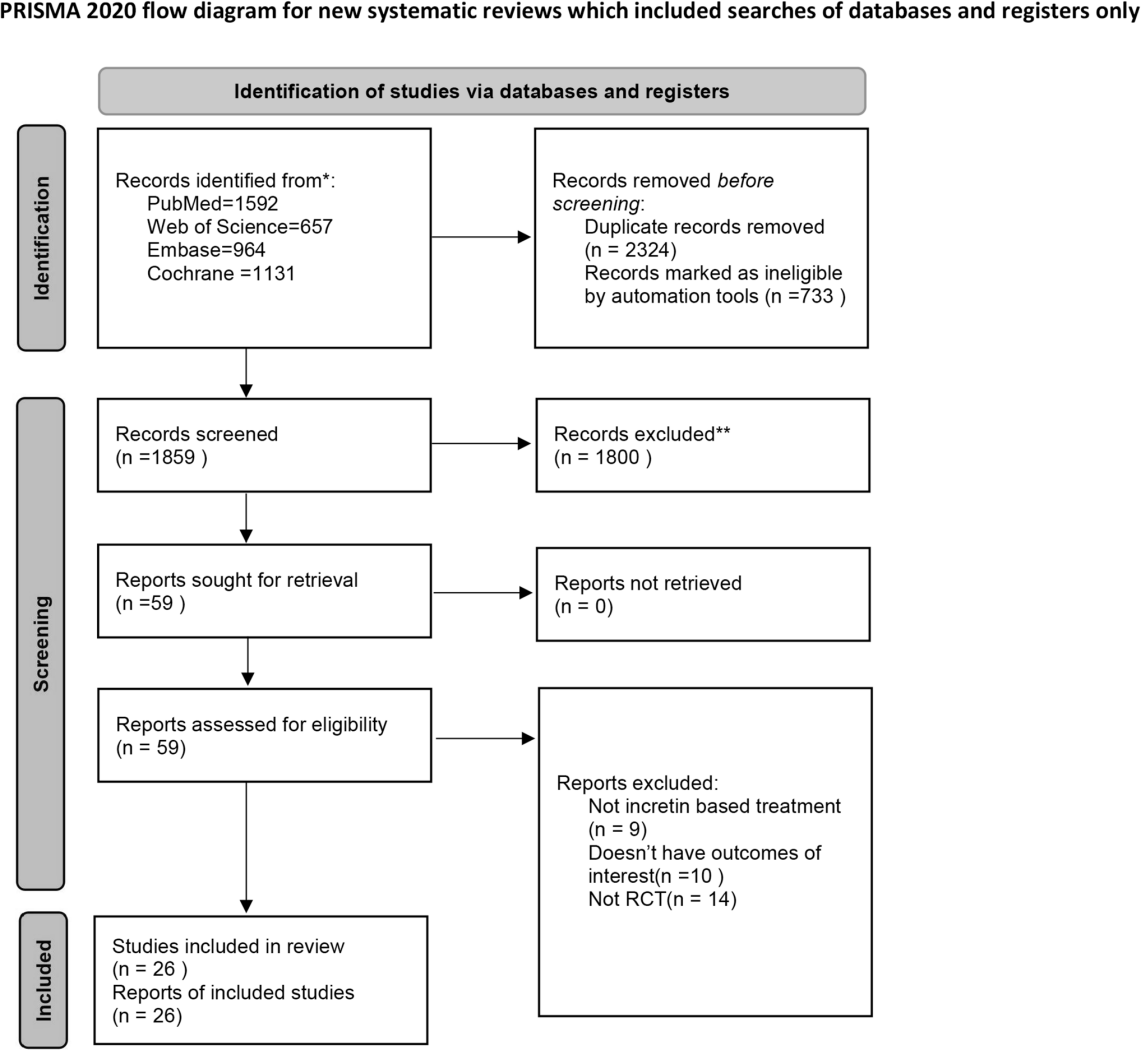
PRISMA Flow Diagram for Study Selection in the Systematic Review and Network Meta-Analysis

A total of 4344 articles were initially retrieved. After duplicate removal in Endnote, 2324 articles remained. Screening by titles and abstracts narrowed this to 59 articles for full-text review. Exclusions were based on non-RCT study design, absence of primary outcomes (weight, FBG, HbA1c), unmet inclusion criteria, or lack of incretin-based dual agonist therapies. Ultimately, 26 articles were included in this network meta-analysis. The screening process is outlined in Figure 1.

### Baseline characteristics of the included studies

A total of 26 articles involving 12,091 patients were included in our analysis. For these patients, their disease characteristics were similar, with baseline HbA1c levels ranging from 7.2 to 8.8%, the BMI from 32.2 to 33.4 kg/m^2^ and mean FBG levels from 151.2 to 161.8 mg/dL. The mean age of the participants in the study was between 56.0 and 60.9 years old. In addition, the gender distribution varied across the studies, but the ratio of men to women was generally balanced. These indicators suggest that the groups from different studies were comparable.

The interventions examined in these articles included various dual and triple agonists, such as Tirzepatide, Cotadutide, Mazdutide and Retatrutide, targeting different receptors like GLP-1/GR and GIP/GLP-1. Additionally, some studies included rapid-acting and long-acting insulin analogues (e.g., Insulin lispro and Insulin Degludec) as well as mono-incretin based therapies like Semaglutide, which were used as comparators. The range of treatment options was demonstrated by the varying dosing regimens and doses, as detailed in Supplementary Table 1.

### Literature quality assessment

The quality of RCTs included in this study was generally rated as “good”. But several factors have led to some downgrades. Specifically, some studies were open-label trials, which may potentially introduce bias [12–15]. Additionally, several studies, including one study by Furihata et al. (2021), did not sufficiently discuss the rationale for their sample size setting, potentially raising concerns regarding the reliability of statistical validity [15–20]. Complete assessment results are detailed in the Attachment 1.

### Results of the network meta-analysis

#### Weight change

**Fig. 2:**
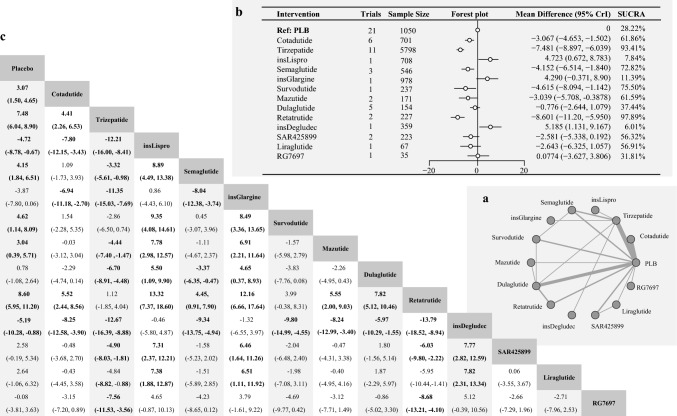
**a** Network Diagram of Treatment Comparisons for Change in Body Weight. This network meta-analysis (NMA) graph illustrates the comparisons of incretin-based therapies, insulins, and other treatments in terms of change in body weight from baseline in T2DM. Each node represents a treatment. Lines between nodes indicate direct comparisons, with line thickness reflecting the number of supporting studies. Treatments not directly linked are compared through indirect evidence within the NMA framework. **b** Forest Plot and SUCRA Rankings for Change in Body Weight. This figure presents the relative effects of treatments on body weight change from baseline. The forest plot displays mean differences (MD) with 95% credible intervals (CrI), comparing each treatment to a reference treatment (Placebo). A negative MD indicates a greater reduction in fasting glucose. The SUCRA (Surface Under the Cumulative Ranking Curve) plot ranks treatments by their probability of being the most effective in reducing body weight, with higher SUCRA values indicating greater weight reduction potential. **c** League Table: Pairwise Comparisons of Treatments for Change in Body Weight. This table summarizes pairwise comparisons of treatment efficacy in terms of body weight change from baseline. Each cell reports the MD with 95% CrI for a given treatment pair. Underlined values indicate direct comparisons derived from head-to-head trials, distinguishing them from indirect comparisons obtained through network meta-analysis

The analysis included 24 studies with 11,254 patients across 14 interventions. The network structure illustrating weight change is shown in Figure 2. According to the heterogeneity and consistency tests using the node-splitting method, this network meta-analysis conformed to the homogeneity and consistency assumptions (Figures S1a and S2).

Retatrutide showed the most significant weight reduction among incretin-based therapies compared to placebo (MD: − 8.601; 95% CrI: − 11.20 to − 5.95, SUCRA: 97.89%). Tirzepatide also demonstrated substantial weight reduction (MD: − 7.481; 95% CrI: − 8.897 to − 6.039, SUCRA: 93.41%). Other effective agents included Survodutide (MD: − 4. 615; 95% CrI: − 8.094 to − 1.142, SUCRA: 75.50%), Semaglutide (MD: − 4.152; 95% CrI: − 6.514 to − 1.840, SUCRA: 72.82%) and Mazdutide (MD: − 3.039; 95% CrI: − 5.708 to − 0.388, SUCRA: 61.59%. Cotadutide also contributed to weight loss (MD: − 3.067; 95% CrI: − 4.653 to − 1.502, SUCRA: 61.86%).

Conversely, other incretin-based therapies, such as Liraglutide, Dulaglutide, SAR425899, and RG7697, did not significantly affect weight. Insulin Glargine was associated with weight gain compared to placebo (MD: 4.290; 95% CrI: 0.371, 8.90, SUCRA: 11.39%), as was Insulin Lispro (MD: 4.723; 95% CrI: 0.672, 8.783, SUCRA: 7.84%) and Insulin Degludec (MD: 5.185; 95% CrI: 1.131, 9.167, SUCRA: 6.01%).

#### FBG

**Fig. 3:**
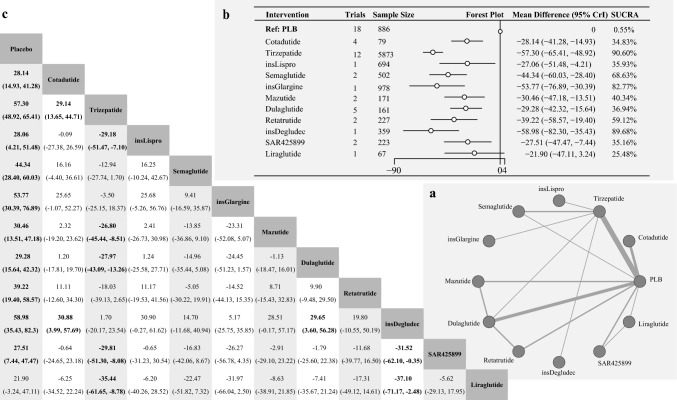
**a** Network Diagram of Treatment Comparisons for Change in Fasting Blood Glucose (FBG). This network meta-analysis (NMA) graph illustrates comparisons of incretin-based therapies, insulins, and other treatments in terms of FBG change from baseline in T2DM. Each node represents a treatment. Lines between nodes indicate direct comparisons, with line thickness reflecting the number of supporting studies. Treatments without direct connections are compared through indirect evidence within the NMA framework. **b** Forest Plot & SUCRA Rankings for Change in FBG. This figure presents the relative effects of treatments on FBG change from baseline. The forest plot displays mean differences (MD) with 95% credible intervals (CrI), comparing each treatment to a reference treatment (placebo). A negative MD indicates a greater reduction in fasting glucose. The SUCRA (Surface Under the Cumulative Ranking Curve) plot ranks treatments by their probability of being the most effective in lowering FBG, with higher SUCRA values indicating greater reductions in fasting glucose levels. **c** League Table: Pairwise Comparisons of Treatments for Change in FBG. This table summarizes pairwise comparisons of treatment efficacy in terms of FBG change from baseline. Each cell reports the MD with 95% CrI for a given treatment pair. Underlined values indicate direct comparisons derived from head-to-head trials, distinguishing them from indirect comparisons obtained through NMA

In terms of FBG, 22 studies were included in the analysis, involving 10,220 patients and covering 12 different interventions. Figure 3 shows the network structure for FBG changes, with tests confirming homogeneity and consistency (Figures S1b and S3).

Specifically, Tirzepatide, a dual GIP and GLP-1 receptor agonist, resulted in the most significant reduction in FBG among all incretin-based therapies. Compared to the placebo, the MD [95% CrI] was − 57.30 [ − 65.41, − 48.92], with a SUCRA of 90.60%, indicating a superior efficacy in lowering FBG levels. Similarly, significant efficacy was observed in Semaglutide, a GLP-1 agonist, (MD [95% CrI] = − 44.34 [ − 60.03, − 28.40], SUCRA = 68.63%). Retatrutide, a triple agonist, also significantly decreased the FBG levels (MD [95% CrI] = − 39.22 [ − 58.57, − 19.40], SUCRA = 59.12%).

According to this study, other incretin-based therapies also exhibited notable efficacy. For example, Mazdutide, a GLP-1/ glucagon receptor dual agonist, could lower FBG levels (MD [95% CrI] = − 30.46 [ − 47.18, − 13.51], SUCRA = 40.34%). Furthermore, Dulaglutide a GLP-1 agonist, had an MD [95% CrI] of − 29.28[ − 42.32, − 15.64] and a SUCRA of 36.94%. Similarly, SAR425899, a dual agonist, showed an MD [95% CrI] of − 27.51 [ − 47.47, − 7.44] with a SUCRA of 35.16%, while Cotadutide, another dual agonist demonstrated an MD [95% CrI] of − 28.14 [ − 41.28, − 14.93] with a SUCRA of 34.83%. In contrast, Liraglutide, a GLP-1 agonist, did not significantly lower FBG levels, indicating that it was not as effective as other incretin-based therapies.

Among non-incretin-based therapies, Insulin Degludec had the highest SUCRA value (89.68%) and markedly lowered FBG levels (MD [95% CrI] = − 58.98 [ − 82.30, − 35. 43]). This significant reduction was also observed in Insulin Glargine (MD [95% CrI] = − 53.77 [ − 76.89, − 30.39], SUCRA = 82.77%). Insulin Lispro also significantly lowered FBG levels, although to a lesser extent (MD [95% CrI] = − 27.06 [ − 51.48, − 4.21], SUCRA = 35.93%).

#### HbA1c

**Fig. 4:**
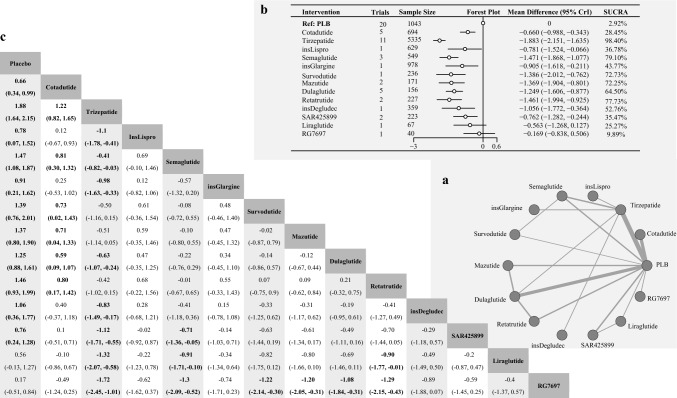
**a** Network Diagram of Treatment Comparisons for Change in HbA1c. This network meta-analysis (NMA) graph illustrates comparisons of incretin-based therapies, insulins, and other treatments in terms of HbA1c change from baseline in T2DM. Each node represents a treatment. Lines between nodes indicate direct comparisons, with line thickness reflecting the number of supporting studies. Treatments without direct connections are compared through indirect evidence within the NMA framework. **b** Forest Plot & SUCRA Rankings for Change in HbA1c. This figure presents the relative effects of treatments on HbA1c change from baseline. The forest plot displays mean differences (MD) with 95% credible intervals (CrI), comparing each treatment to a reference treatment (placebo). A negative MD indicates a greater reduction in fasting glucose. The SUCRA (Surface Under the Cumulative Ranking Curve) plot ranks treatments by their probability of being the most effective in lowering HbA1c, with higher SUCRA values indicating greater glycemic control. **c** League Table: Pairwise Comparisons of Treatments for Change in HbA1c. This table summarizes pairwise comparisons of treatment efficacy in terms of HbA1c change from baseline. Each cell reports the MD with 95% CrI for a given treatment pair. Underlined values indicate direct comparisons derived from head-to-head trials, distinguishing them from indirect comparisons obtained through NMA

In terms of HbA1c levels, 24 studies were included in the analysis, involving 10,707 patients and covering 14 different interventions. Figure 4 depicts the network structure illustrating changes in HbA1c level. According to the heterogeneity and consistency tests using the node-splitting method, this network meta-analysis conformed to the homogeneity and consistency assumptions (Figures S1c and S4).

Tirzepatide demonstrated the most significant decrease in HbA1c levels across all therapies, including both incretin-based therapies and insulin, when compared with placebo (MD [95% CrI] = − 1.883 [ − 2.151, − 1.635], SUCRA = 98.40%). Semaglutide also exhibited notable efficacy (MD [95% CrI] = − 1.471 [ − 1.868, − 1.077], SUCRA = 79.10%), followed closely by Retatrutide, which achieved a comparable reduction in HbA1c levels (MD [95% CrI] = − 1.461 [ − 1.994, − 0.925], SUCRA = 77.73%).

This study also identified other effective incretin-based therapies. Specifically, the MD [95% CrI] for Survodutide was − 1.386 [ − 2.012, − 0.762] with a SUCRA of 72.73%, while for Mazdutide, the MD [95% CrI] was − 1.369 [ − 1.904, − 0.801] with s SUCRA of 72.25%. Dulaglutide showed an MD [95% CrI] of − 1.249 [ − 1.606, − 0.877] and a SUCRA of 64.50%. In contrast, some incretin-based therapies demonstrated lower efficacy, as indicated by their MD [95% CrI] and SUCRA values. Results for these therapies are outlined below: SAR425899 (a dual glucagon receptor/GLP-1 agonist): MD [95% CrI] = − 0.762 [ − 1.282, − 0.244], SUCRA = 35.47%; Cotadutide: MD [95% CrI] = − 0.660 [ − 0.988, − 0.343], SUCRA = 28.45%; Liraglutide: MD [95% CrI] = − 0.563 [ − 1.268, 0.127], SUCRA = 25.27%; and RG7697: MD [95% CrI] = − 0.169 [ − 0.838, 0.506], SUCRA = 9.89%. Among other non-incretin-based therapies, Insulin Degludec resulted in a notable decrease in HbA1c levels (MD [95% CrI] = − 1.056 [ − 1.772, − 0.364], SUCRA = 52.76%). Results for other similar therapies are as follows: Insulin Lispro: MD [95%CrI] = − 0.781 [ − 1.524, − 0.066], SUCRA = 36.78%; and Insulin Glargine: MD [95% CrI] = − 0.905 [ − 1.618, − 0.211], SUCRA = 43.77%.

#### Safety

The analysis showed that both Tirzepatide (RR [95% CrI] = 1.149 [1.039, 1.333], SUCRA = 43.97%) and Cotadutide (RR [95% CrI] = 1.379 [1.159, 1.682], SUCRA = 13.53%) led to a statistically significant increase in the risk of AEs compared with placebo, as presented in Figure S7. However, the risk of SAEs was not associated with any of the medications in the study. Additionally, Semaglutide (RR [95% CrI] = 0.3494 [0.1264, 0.7805], SUCRA = 93.40%) considerably lowered the risk of SAEs compared with the placebo, as shown in Figure S8. Heterogeneity and inconsistency tests confirmed the analysis met homogeneity and consistency assumptions, supporting the reliability of the findings (Figures S1d, S1e, S5, S6).

#### Influence of different dosages and treatment duration

Many treatments in this analysis were evaluated at varying doses and treatment durations. In our stratified analysis, we found that even when accounting for dose and duration, the overall ranking of efficacy and safety remained largely consistent in most cases.

Notably, Retatrutide exhibited a trend of increasing efficacy with higher doses and longer treatment durations in weight reduction and HbA1c lowering, suggesting a dose-response and duration-dependent effect.

A detailed analysis of the impact of different dosages and treatment durations, along with the corresponding results, is presented in Supplementary Figures S9–S16.

## Discussion

### Advantages and similarities of Retatrutide and Tirzepatide

Both Retatrutide and Tirzepatide demonstrated notable improvements in weight loss and glycemic control, primarily due to their ability to target multiple metabolic pathways. As a dual agonist, Tirzepatide mainly targets GIP and GLP-1 receptors, which play a key role in regulating insulin secretion, glucose metabolism and appetite [7]. Tirzepatide’s higher affinity for GIP receptors enhances its effect in lowering blood glucose levels and promoting weight loss, which is similar to the action of Retatrutide [21]. Although Retatrutide provided additional benefits through glucagon receptor activation [22], the differences in weight loss and glycemic control between Retatrutide and Tirzepatide were not significant. This could be attributed to different mean treatment durations in the included studies (38.5 weeks for Tirzepatide and 18 weeks for Retatrutide), or the small number of studies included in the analysis. Furthermore, the primary mechanisms driving these outcomes in Retatrutide’s action may be the GLP-1 and GIP pathways [23, 24].

### Lower efficacy of RG7697 compared to Tirzepatide

Initially, it was believed that RG7697 belonged to the same class as Tirzepatide and would be equally effective in weight loss and glycemic control. However, this network meta-analysis showed different results. This may be because, although RG7697 is a dual GIP/GLP-1 agonist like Tirzepatide, shows a more balanced receptor affinity in its interaction with the GIP and GLP receptor [25, 26]. In contrast, Tirzepatide has a higher affinity for the GIP receptor than GLP-1, a difference that could be crucial for achieving notable metabolic changes, such as improved insulin secretion, better glycemic control and more significant weight loss [27]. Challenges in GIP receptor agonist development may arise from inherent differences between human and mouse GIP receptors, complicating the transformation of preclinical findings to human efficacy [28]. Tirzepatide, with its optimized receptor binding properties, overcomes these challenges, which may also account for its advantage in clinical practice compared to RG7697 [25, 29].

### Similar efficacy of Semaglutide and GLP-1/glucagon receptor agonists

Semaglutide, a potent GLP-1 receptor agonist, demonstrated comparable efficacy with dual GLP-1/glucagon receptor agonists. This is largely attributed to its potent and sustained activation of GLP-1 receptors, which is essential for regulating blood sugar levels and suppressing appetite [10, 30]. The structural modifications of Semaglutide, such as introducing isobutyric acid (Aib) at the second N-terminal residue and acylation of lysine at the 20th N-terminal residue, enhance its resistance to DPP-4 degradation, prolong its half-life through binding to plasma albumin, and thereby achieve sustained GLP-1 receptor activation and superior efficacy [31]. Clinical comparisons revealed that Semaglutide achieve similar outcomes in weight loss and glycaemic control as dual GLP-1/glucagon receptor agonists, indicating that its targeted GLP-1 activation alone is sufficient for effective metabolic management [32]. These findings suggest that the single-receptor agonist like Semaglutide can perform as well as, and in some cases may exceed, dual-receptor agonists in treating T2DM.

### Safety

This network meta-analysis concluded that Cotadutide and Tirzepatide exhibited a higher risk of AEs compared with the placebo. Cotadutide targets GLP-1 and glucagon receptors, which may lead to gastrointestinal side effects similar to those commonly observed with GLP-1 agonists, such as nausea and vomiting [16, 33]. Similarly, Tirzepatide, by activating both GIP and GLP-1 receptors also increased the incidence of gastrointestinal side effects. However, these adverse events were generally mild and did not significantly increase the risk of SAEs [34, 35]. On the other hand, Semaglutide, a GLP-1 receptor agonist, exhibited a lower risk of SAEs. This reflects its established safety profile, with fewer serious side effects, and may help prevent the occurrence SAEs, which may be related to its specific GLP-1 receptor activation mechanism [36].

### Strengths and limitations

As the most comprehensive and up-to-date systematic review and network meta-analysis specifically designed to compare the dual and triple-incretin based agonists for managing T2DM, this study makes several unique contributions to the field. Most previous studies have either focused on GLP-1 receptor-specific agonists and polyagonists for weight management in individuals with obesity or overweight without diabetes, or, as in the case of the recent meta-analysis by Yao et al. (2024), has evaluated GLP-1 receptor agonists for T2DM in a broader sense. Conversely, this analysis focuses specifically on dual and triple incretin-based therapies that combine GLP-1 with additional receptors, such as GIP and GCGR [8–10]. Furthermore, it encompasses a broader range of dual therapies, such as GLP-1/GCGR agonists (e.g., SAR425899, Cotadutide) and GLP-1/GIP agonists (e.g., RG7697), along with standard treatments like insulins. By examining these multi-receptor therapies, this study explores how specific receptor targeting and varying receptor affinities can enhance clinical efficacy. This tailored approach allows treatment to be customized by aligning receptor activation with the unique metabolic, glycemic and safety needs of individual patients.

However, certain limitations are inevitable in this study. First, the robustness and generalizability of the results may be limited by the small number of original studies it includes. Second, the small number of studies made it impossible to perform a thorough evaluation of publication bias, which might have affected the reliability of the findings. Third, there is limited supporting data on GIP-targeting medications. Additionally, the shorter treatment durations in some studies, particularly for Retatrutide, may mean that the analysis did not fully capture its potential long-term benefits for complex metabolic needs. Another challenge is the absence of direct comparison data among different glucagon-like co-agonists.

Furthermore, while this study primarily focused on dual and triple incretin-based agonists, the structure of the network meta-analysis led to the inclusion of some non-multi-incretin-based interventions (e.g., mono-incretin agonists and insulin therapies) to facilitate indirect comparisons. Our inclusion criteria required incorporating all RCTs evaluating dual and triple incretin-based agonists, even if their comparator arms included mono-incretin therapies or other standard treatments. While this approach may enhance indirect comparisons where direct head-to-head trials are lacking, we acknowledge that the inclusion of these additional interventions could introduce some degree of heterogeneity. Despite the overall consistency between direct and network comparisons in inconsistency testing and heterogeneity analysis, the evidence derived from direct comparisons remains relatively limited, which limits the ability to draw detailed conclusions about their relative efficacy. Finally, variations in the study design, patient population and intervention protocols may introduce heterogeneity and diminish the comparability of the results.

### Practical clinical implications and future research directions

The findings from this study highlight the potential of incretin-based dual and triple agonists in personalizing T2DM treatment through receptor-specific targeting. By focusing on combinations of GLP-1, GIP, and glucagon receptors, these therapies allow clinicians to align treatment strategies with individual patient profiles, optimizing both efficacy and safety.

#### Clinical applications for specific patient profiles

Based on this network meta-analysis, the following treatment recommendations are proposed for specific patient needs.

For T2DM patients with obesity, where both glucose regulation and weight management are primary therapeutic goals. Tirzepatide and Retatrutide offer effective treatment options. While no significant difference in efficacy was observed between these agents, the shorter treatment duration for Retatrutide in this study may have influenced these findings. Both agents can be considered depending on the patient’s broader treatment goals and metabolic profile.

Tirzepatide, with its dual and balanced activation of GLP-1 and GIP receptors, enhances insulin secretion, supports glucose control, and promotes in weight reduction, making it highly beneficial for patients for whom weight loss is a key therapeutic goal. In contrast, Retatrutide includes additional activation of the glucagon receptor, which, while not translating into additional short-term efficacy benefits in this study, the glucagon receptor activation may provide broader metabolic advantages over a longer duration. In clinical practice, this means that if a patient’s primary goals are short-term blood glucose control and weight loss, Tirzepatide may be as effective as Retatrutide without the added glucagon receptor activation. However, for patients with complex metabolic needs, such as those at risk for non-alcoholic fatty liver disease (NAFLD) or dyslipidaemia, Retatrutide could be a valuable option - especially as future long-term studies may reveal additional benefits from glucagon receptor activation.

Despite the demonstrated efficacy of co-agonists, Semaglutide remains advantageous for general T2DM patients due to its efficacy and favourable safety profile, especially for those prioritizing HbA1c management without the need for substantial weight-loss support. Semaglutide’s low risk of SAEs makes it particularly suitable for patients sensitive to side effects or those who may benefit from potential cardiovascular benefits [37].

However, it is important to emphasize that due to the lack of direct comparison evidence, the findings of this study should still be considered preliminary, and caution should be exercised when applying them in clinical practice. While network meta-analysis enables a comprehensive evaluation of multiple therapies, it does not fully replace direct head-to-head RCTs. The inclusion of non-multi-incretin-based interventions in our analysis helped strengthen indirect comparisons, but future research with direct comparisons will be essential to further validate these findings.

#### Safety considerations in therapy selection

The distinct safety profiles of these therapies highlight the importance of personalized patient monitoring. This study indicates that Tirzepatide and Cotadutide are associated with a increase in overall AEs, underscoring the need for careful monitoring when initiating these therapies [16, 35]. Given these, clinicians may benefit from closely monitoring patients for AEs during the early stages of treatment, particularly those who may be prone to GI-related side effects.

#### Future research directions - Receptor affinity and Long-term outcomes

While this study provides valuable initial insights into receptor-specific therapies for T2DM management, further research is essential to confirm and build upon these findings. Long-term randomized trials are essential to evaluate the sustained efficacy, safety, and impact of dual and triple agonists on cardiovascular and broader metabolic outcomes [38, 39].

Retatrutide, with its additional activation of the glucagon receptor, may offer added benefits in lipid metabolism and fat utilization over longer treatment periods. The lack of significant efficacy differences between Retatrutide and Tirzepatide observed in this study may be result from the shorter treatment duration for Retatrutide. The potential metabolic benefits from glucagon receptor activation may require longer treatments to fully emerge. Future long-term studies on Retatrutide and similar agents will be essential to clarify their role in managing complex metabolic conditions.

The differences observed in efficacy between Tirzepatide and RG7697 - despite both targeting GLP-1 and GIP receptors, highlight the importance of receptor affinity and balance in therapeutic outcomes. Research focused on optimizing receptor affinity ratios for GLP-1, GIP, and glucagon receptor could lead to new therapeutic combinations that maximize benefits for specific patient subgroups. Comparative studies examining how receptor combinations and affinities influence glycemic control, weight management, and other metabolic health markers are essential to support the development of more targeted and effective T2DM therapies.

As network meta-analysis results cannot replace direct head-to-head comparisons, future studies directly comparing different incretin-based multi-receptor therapies will be necessary to further validate the conclusions of this study.

## Conclusion

This study highlights the potential of dual and triple incretin-based therapies to personalize T2DM treatment through receptor-specific targeting, enabling clinicians to better align treatments with individual patient needs for improved efficacy and safety. Tirzepatide and Retatrutide show promise for T2DM patients with obesity, supporting both glycemic control and weight management. Tirzepatide’s dual GLP-1 and GIP activation is effective for weight loss and glycemic control, while Retatrutide’s added glucagon receptor activation may offer broader metabolic benefits for patients with complex needs. Despite the efficacy of co-agonists, Semaglutide remains advantageous for the general T2DM population, providing reliable glycemic control with a favourable safety profile, especially for patients focused on HbA1c management without major weight-loss needs. Long-term studies are needed to confirm these findings and refine receptor-targeted therapies, advancing patient-centred diabetes care.

## Novelty Statement

- What is already known?

GLP-1 receptor agonists effectively manage glycemic control and weight loss in T2DM, and dual/triple incretin agonists targeting GLP-1, GIP, and Glucagon-receptor show promise but lack receptor-specific evaluations.

- What this study has found?

Tirzepatide, with its balanced GLP-1/GIP receptor activation, is the most effective for weight loss and glycemic control, particularly in obese patients. Retatrutide’s added glucagon receptor activation may offer broader metabolic benefits for patients with complex needs. Semaglutide remains advantageous for general T2DM management, providing reliable glycemic control with a favourable safety profile for HbA1c-focused patients.

- Implications: Receptor-specific therapies support personalized T2DM care; However, robust head-to-head trials with multiple treatment arms (e.g., RCTs or pragmatic trials) are needed to strengthen comparative effectiveness evidence.

## Supplementary Information

Below is the link to the electronic supplementary material.

Supplementary file1 (DOCX 31 KB)

Figure S1 Inconsistency Test Results for Weight Change, FBG, HbA1c, AEs, and SAEs Across Incretin-Based Therapies and Comparators in T2DM. Supplementary file2 (TIF 428 KB)

Figure S2 Heterogeneity Test Results for Changes in Weight. Supplementary file3 (TIF 1442 KB)

Figure S3 Heterogeneity Test Results for Changes in Fasting Blood Glucose. Supplementary file4 (TIF 954 KB)

Figure S4 Heterogeneity Test Results for Changes in HbA1c. Supplementary file5 (TIF 1101 KB)

Figure S5 Heterogeneity Test Results for Changes in Adverse Event. Supplementary file6 (TIF 167 KB)

Figure S6 Heterogeneity Test Results for Changes in Serious Adverse Event. Supplementary file7 (TIF 305 KB)

Figure S7 Netplot, Forest Plot, and League Table of Adverse Events Risk Across Incretin-Based Therapies, Insulins, and Their Comparators in T2DM. Supplementary file8 (TIF 767 KB)

Figure S8 Netplot, Forest Plot, and League Table of Serious Adverse Events Risk Across Incretin-Based Therapies, Insulins, and Their Comparators in T2DM. Supplementary file9 (TIF 797 KB)

Figure S9 Impact of Dosage and Treatment Duration on Weight Change. Supplementary file10 (PDF 119 KB)

Figure S10 Impact of Dosage and Treatment Duration on FBG Reduction. Supplementary file11 (PDF 147 KB)

Figure S11 Impact of Dosage and Treatment Duration on HbA1c Reduction. Supplementary file12 (PDF 133 KB)

Figure S12 Forest Plot for Impact of Dosage and Treatment Duration on FBG, HbA1C, Weight. Supplementary file13 (TIF 4485 KB)

Figure S13 Inconsistency Tests Results for Dosage and Treatment Duration Change on FBG, HbA1C, Weight. Supplementary file14 (TIF 2506 KB)

Figure S14 Heterogeneity Test Results for Dosage and Treatment Duration FBG Reduction. Supplementary file15 (TIF 16575 KB)

Figure S15 Heterogeneity Test Results for Dosage and Treatment Duration on HbA1C Reduction. Supplementary file16 (TIF 11257 KB)

Figure S16 Heterogeneity Test Results for Dosage and Treatment Duration on Weight Change. Supplementary file17 (TIF 7768 KB)

Attachment 1 Quality Assessment of Included Studies Using the NIH Quality Assessment Tool. Supplementary file18 (DOCX 21 KB)

Supplementary file19 (DOCX 32 KB)

## Data Availability

The datasets used and analysed during the current study are available from the corresponding author upon reasonable request.
