# Supplementary material for: Beyond GLP-1: efficacy and safety of dual and triple incretin agonists in personalized type 2 diabetes care—a systematic review and network meta-analysis
Source: Acta Diabetol. 2025 Jun 5;62(9):1359–70. doi: 10.1007/s00592-025-02534-y (PMC12433336; doi:10.1007/s00592-025-02534-y)
Supplement: Supplementary file 1 — Supplementary file1 (DOCX 31 KB) [file 592_2025_2534_MOESM1_ESM.docx]

**Supplementary Table 1** Baseline Characteristics of Included Studies

| **Author** | **Year** | **Intervention** | **Targeted Receptors and**  **Drug Class** | **Sample  size** | **Sex**  **(F, M)** | **Age**  **(Mean, SD)** | **Weight**  **(kg)** | **BMIb**  **(kg/m²)** | **HbA1c**  **(%)** | **Fasting  Serum  Glucose (mg/dL)** | **NCT** |
| --- | --- | --- | --- | --- | --- | --- | --- | --- | --- | --- | --- |
| Ambery P et al. | 2018 | MEDI0382  (Cotadutide) | GLP-1, GR  Dual Agonist | 25 | 13, 12 | 56.00, 7.20 | 95.90, 18.90 | 32.20, 4.40 | 7.20, 0.60 | 153, 34.20 | NCT02548585 |
|  |  | PLB | N/A | 26 | 15, 11 | 56.90, 6.20 | 99.90, 15.10 | 33.40, 3.40 | 7.30, 0.70 | 151.2, 32.40 |  |
| Rosenstock J et al. | 2023 | Tirzepatide | GIP, GLP-1  Dual Agonist | 717 | 426, 291 | 56.90, 6.20 | 90.70, 18.50 | 33.30, 5.40 | 8.80, 0.99 | 158.4, 56.40 | NCT04537923 |
|  |  | Insulin Lispro | Short-acting Insulin | 708 | 396, 312 | 59.00, 9.70 | 90.30, 17.70 | 33.00, 5.20 | 8.80, 0.96 | 156.3, 56.10 |  |
| Dahl D et al. | 2022 | Tirzepatide | GIP, GLP-1  Dual Agonist | 355 | 157, 198 | 60.99, 10.00 | 95.53 21.61 | 33.47, 5.99 | 8.30, 0.86 | 161.82, 53.2341 | NCT04039503 |
|  |  | PLB | N/A | 120 | 54,66 | 60.00, 10.00 | 94.10, 21.80 | 33.20, 6.30 | 7.34, 1.57 | 164.1, 45.0 |  |
| Rosenstock J et al. | 2021 | Tirzepatide | GIP, GLP-1  Dual Agonist | 363 | 172, 191 | 54.27, 11.59 | 86.20, 19.72 | 31.97, 6.74 | 7.91,0.88 | 153.09, 39.7341 | NCT03954834 |
|  |  | PLB | N/A | 478 | 231, 247 | 53.60, 12.80 | 84.80, 20.00 | 31.70, 6.10 | 8∙05, 0∙80 | 154.8, 40.3 |  |
| Heise T et al. | 2022 | Tirzepatide | GIP, GLP-1  Dual Agonist | 45 | 14, 31 | 61.10, 7.10 | 94.15, 14.00 | 31.28, 5.01 | 7.83, 0.72 | 139.3, 30.2 | NCT03951753 |
|  |  | Semaglutide | GLP-1 Receptor Agonist | 44 | 10, 34 | 63.70, 5.90 | 92.65, 14.01 | 30.82, 3.84 | 7.70, 0.60 | 128.6, 25.0 |  |
|  |  | PLB | N/A | 28 | 7, 21 | 60.40, 7.60 | 98.74, 14.61 | 32.24, 3.96 | 7.90, 0.51 | 126.6, 23.6 |  |
| Frías et al. | 2021 | Tirzepatide | GIP, GLP-1  Dual Agonist | 1409 | 752, 657 | 56.47, 10.31 | 93.70, 22.11 | 34.20, 6.86 | 8.29, 1.03 | 173.1993, 52.0137 | NCT03987919 |
|  |  | Semaglutide | GLP-1 Receptor Agonist | 469 | 244, 225 | 56.90, 10.80 | 93.70, 21.12 | 34.20, 7.15 | 8.25,1.01 | 171.4, 49.77 |  |
| Del Prato S et al. | 2021 | Tirzepatide | GIP, GLP-1  Dual Agonist | 997 | 387, 610 | 63.44, 8.67 | 90.30, 18.34 | 32.63, 5.54 | 8.54, 0.91 | 173.97, 51.65 | NCT03730662 |
|  |  | Insulin Glargine | Long-acting Insulin | 1005 | 364, 641 | 63.80, 8.50 | 90.20, 19.00 | 32.50, 5.55 | 8.50, 0.85 | 168.40, 49.72 |  |
| Garvey WT et al. | 2023 | Tirzepatide | GIP, GLP-1  Dual Agonist | 623 | 317, 306 | 53.95, 10.65 | 100.25, 20.50 | 35.85, 6.25 | 8.0349, 0.9179 | 160.20, 45.97 | NCT04657003 |
|  |  | PLB | N/A | 315 | 159, 156 | 54.70, 10.50 | 101.70, 22.30 | 36.60,7.30 | 7·89, 0·84 | 158.4 , 46.8 |  |
| Golubic R et al. | 2023 | MEDI0382 (Cotadutide) | GLP-1, GR  Dual Agonist | 19 | 1, 18 | 59.50, 8.40 | 99.30, 10.80 | 32.20, 2.20 | 7.00, 0.46 | 154.20, 13.20 | NCT03596177 |
|  |  | PLB | N/A | 9 | 2,7 | 62.20, 7.20 | 101.40, 16.00 | 34.50, 2.80 | 6.73, 0.37 | 135.72, 10.62 |  |
| Blüher M et al. | 2023 | BI 456906 (survodutide) | GLP-1, GR  Dual Agonist | 302 | 134, 168 | 57.52, 9.52 | 97.23, 21.88 | 34.02, 6.01 | 8.06, 0.85 | 184.62, 24.40 | NCT04153929 |
|  |  | Semaglutide | GLP-1 Receptor Agonist | 50 | 16， 34 | 55.80, 10.50 | 96.70, 20.00 | 33.40, 6.10 | 8.03, 0.84 | 183.76, 24.11 |  |
|  |  | PLB | N/A | 59 | 28, 31 | 57.50, 10.50 | 93.00, 21.00 | 33.40, 5.90 | 8.15, 0.84 | 186.31, 24.11 |  |
| Frías JP et al. | 2020 | Tirzepatide | GIP, GLP-1  Dual Agonist | 85 | 31, 54 | 57.81, 8.72 | 88.76, 17.30 | 31.70, 4.98 | 8.39, 1.06 | 186.50, 67.86 | NCT03311724 |
|  |  | PLB | N/A | 26 | 14,12 | 56.00, 10.13 | 89.60, 23.70 | 32.50, 5.70 | 8.23, 1.22 | 168.50, 62.06 |  |
| Jiang H et al. | 2022 | IBI362 (Mazutide) | GLP-1, GR  Dual Agonist | 24 | 10, 14 | 53.70, 9.34 | 68.77, 11.14 | 25.27, 2.51 | 8.73, 0.92 | 205.80, 35.30 | NCT04466904 |
|  |  | Dulaglutide | GLP-1 Receptor Agonist | 6 | 0, 6 | 50.70, 4.80 | 75.10, 10.20 | 26.60, 3.00 | 8.30, 1.40 | 201.60, 55.80 |  |
|  |  | PLB | N/A | 12 | 6,6 | 50.20, 8.50 | 68.20, 11.50 | 26.50, 2.70 | 8.30, 0.70 | 201.60, 46.80 |  |
| Parker VE et al. | 2020 | MEDI0382 (Cotadutide) | GLP-1, GR  Dual Agonist | 46 | 17,29 | 60.09, 7.61 | 94.21, 14.10 | 31.32, 3.47 | 7.35, 0.58 | 160.51, 36.17 | NCT03244800 |
|  |  | PLB | N/A | 19 | 5, 14 | 60.23, 6.78 | 93.77, 17.88 | 31.47, 3.61 | 7.28, 0.59 | 159.35, 29.33 |  |
| Zhang B et al. | 2024 | IBI362 (Mazutide) | GLP-1, GR  Dual Agonist | 149 | 55,94 | 53.98, 11.41 | 76.59, 15.42 | 27.51, 4.04 | 8.05, 0.89 | 169.18, 39.73 | NA |
|  |  | Dulaglutide | GLP-1 Receptor Agonist | 50 | 24, 26 | 52.80, 12.70 | 72.30, 12.80 | 26.70, 3.20 | 7.95, 0.92 | 156.60, 36.00 |  |
|  |  | PLB | N/A | 51 | 23, 28 | 52.60, 11.10 | 73.90, 14.40 | 27.50, 3.50 | 8.16, 0.91 | 165.60, 39.60 |  |
| Rosenstock J et al. | 2023 | LY3437943  Retatrutide | GIP, GLP-1, GR Triple Agonist | 190 | 100, 90 | 56.18, 9.20 | 98.56, 21.51 | 34.96, 6.38 | 8.26, 1.11 | 171.49, 54.69 | NCT04867785 |
|  |  | Dulaglutide | GLP-1 Receptor Agonist | 46 | 33, 13 | 54.90, 10.40 | 100.30, 23.40 | 36.60, 6.80 | 8.23, 0.92 | 151.20, 39.60 |  |
|  |  | PLB | N/A | 45 | 23, 22 | 57.60 , 10.80 | 94.60, 16.60 | 33.80, 4.90 | 8.39, 1.14 | 183.60, 61.20 |  |
| Urva S et al. | 2022 | LY3437943 (Retatrutide) | GIP, GLP-1, GR Triple Agonist | 52 | 24, 28 | 58.09, 7.76 | 86.23, 16.81 | 32.24, 4.94 | 8.63, 0.89 | 192.01, 47.04 | NCT04143802 |
|  |  | Dulaglutide | GLP-1 Receptor Agonist | 5 | 1, 4 | 59.80, 7.50 | 84.90, 14.10 | 30.10, 2.00 | 8·50, 0·86 | 167.40, 25.76 |  |
|  |  | PLB | N/A | 15 | 12, 3 | 58.80, 6.40 | 84.10, 19.90 | 32.30, 6.20 | 8·83, 1·06 | 181.80, 48.10 |  |
| Ludvik B et al. | 2021 | Tirzepatide | GIP, GLP-1  Dual Agonist | 1077 | 488, 589 | 57.37, 9.99 | 94.37, 19.91 | 33.57, 6.06 | 8.19, 0.90 | 170.04, 47.08 | NCT03882970 |
|  |  | Insulin Degludec | ultralong-acting insulin | 360 | 147, 213 | 57.50, 10.10 | 94.00, 20.60 | 33.40, 6.10 | 8·12, 0·94 | 166.68, 41.94 |  |
| Asano M et al. | 2022 | MEDI0382 (Cotadutide) | GLP-1, GR  Dual Agonist | 12 | 5, 7 | 58.50, 8.75 | 77.05, 5.63 | 27.19, 2.38 | 7.41, 0.67 | 165.97, 19.23 | NCT04208620 |
|  |  | Placebo | N/A | 4 | 3, 1 | 60.00, 4.25 | 79.50, 2.68 | 28.05, 1.49 | 7.95, 0.45 | 181.47, 12.92 |  |
| Tillner J et al. | 2018 | SAR425899 | GLP-1, GR  Dual Agonist | 27 | 28, 8 | 58.90, 8.70 | 98.00, 15.35 | 32.00, 3.00 | 7.17, 0.57 | 154.00, 22.50 | NCT02411825 |
|  |  | PLB | N/A | 9 |  |  | 100.20, 8.54 | 32.72, 5.12 | 7.90, 1.56 | 180.00, 35.45 |  |
| Frias JP et al. | 2018 | Tirzepatide | GIP, GLP-1  Dual Agonist | 211 | 96, 115 | 56.96, 8.64 | 91.95, 21.41 | 32.65, 5.92 | 8.18, 1.02 | 166.51, 46.30 | NCT03131687 |
|  |  | Dulaglutide | GLP-1 | 54 | 30, 24 | 58.70, 7.80 | 89.80, 16.90 | 32.40, 5.40 | 8.10, 1.10 | 178.20, 64.80 |  |
|  |  | PLB | N/A | 51 | 22, 29 | 56.60, 8.90 | 91.50, 23.10 | 32.40, 6.00 | 8.00, 0.90 | 163.80, 41.10 |  |
| Feng P et al. | 2023 | Tirzepatide | GIP, GLP-1  Dual Agonist | 20 | 10, 10 | 56.30, 5.19 | 66.30, 7.61 | 25.55, 2.33 | 7.95, 1.15 | 181.47, 33.00 | NCT04235959 |
|  |  | PLB | N/A | 4 | 1, 3 | 56.50, 7.50 | 71.30, 7.10 | 26.4, 1.80 | 7.80, 0.70 | 177.16, 20.09 |  |
| Parker VER et al. | 2022 | MEDI0382 (Cotadutide) | GLP-1, GR  Dual Agonist | 21 | 9, 12 | 71.10, 7.40 | 94.70, 17.60 | 32.4, 4.10 | 7.82, 0.70 | 167.40, 27.00 | NCT03550378 |
|  |  | PLB | N/A | 20 | 11,9 | 70.90, 4.70 | 91.60, 15.80 | 32.90, 5.50 | 7.91, 1.30 | 178.20, 50.40 |  |
| Schiavon M et al. | 2021 | SAR425899 | GLP-1, GR  Dual Agonist | 196 | 92, 104 | 55.44, 9.85 | 103.57, 14.72 | 33.85, 4.81 | 8.03, 0.89 | 183.82, 25.51 | NCT02973321 |
|  |  | Liraglutide | GLP-1 Receptor Agonist | 67 | 36, 31 | 56.10, 11.40 | 104.83, 168.87 | 34.23, 5.51 | 8.11, 0.86 | 186.06, 24.66 |  |
|  |  | PLB | N/A | 33 | 15, 18 | 56.20, 9.30 | 98.22, 13.51. | 32.07, 4.41 | 8.04, 0.86 | 184.05, 24.66 |  |
| Furihata K et al. | 2021 | Tirzepatide | GIP, GLP-1  Dual Agonist | 39 | 1, 38 | 57.40, 8.24 | 74.38, 9.88 | 26.08, 3.02 | 8.03, 0.76 | 172.18, 28.23 | NCT03322631 |
|  |  | PLB | N/A | 9 | 0, 9 | 57.40, 11.60 | 63.00, 7.80 | 22.60, 2.10 | 7.80, 0.90 | 173.90, 33.10 |  |
| Nahra R et al. | 2021 | MEDI0382 (Cotadutide) | GLP-1, GR  Dual Agonist | 612 | 333, 279 | 56.93, 10.03 | NR | NR | NR | NR | NCT03235050 |
|  |  | Liraglutide | GLP-1 Receptor Agonist | 110 | 60, 50 | 55.50, 9.80 | NR | NR | NR | NR |  |
|  |  | PLB | N/A | 112 | 55, 57 | 57.30, 9.50 | NR | NR | NR | NR |  |
| Schmitt C et al. | 2017 | RG7697 | GIP, GLP-1  Dual Agonist | 42 | 18, 24 | 54.55, 7.85 | 95.55, 18.27 | 33.36, 3.69 | 7.76, 1.08 | 157.08, 32.03 | NCT01789788 |
|  |  | PLB | N/A | 14 | 6,8 | 50.30, 8.00 | 100.60, 13.60 | 33.70, 4.20 | 7.71, 1.11 | 153.90, 25.02 |  |

Abbreviations: GLP-1, glucagon-like peptide-1; GR, glucagon receptor; GIP, glucose-dependent insulinotropic polypeptide; PLB, placebo; N/A, not applicable; SD, standard deviation; BMI, body mass index; HbA1c, glycated hemoglobin; F, female; M, male; NCT, national clinical trial number.
