# Supplementary figures and images for: Beyond GLP-1: efficacy and safety of dual and triple incretin agonists in personalized type 2 diabetes care—a systematic review and network meta-analysis
Source: Acta Diabetol. 2025 Jun 5;62(9):1359–70. doi: 10.1007/s00592-025-02534-y (PMC12433336; doi:10.1007/s00592-025-02534-y)

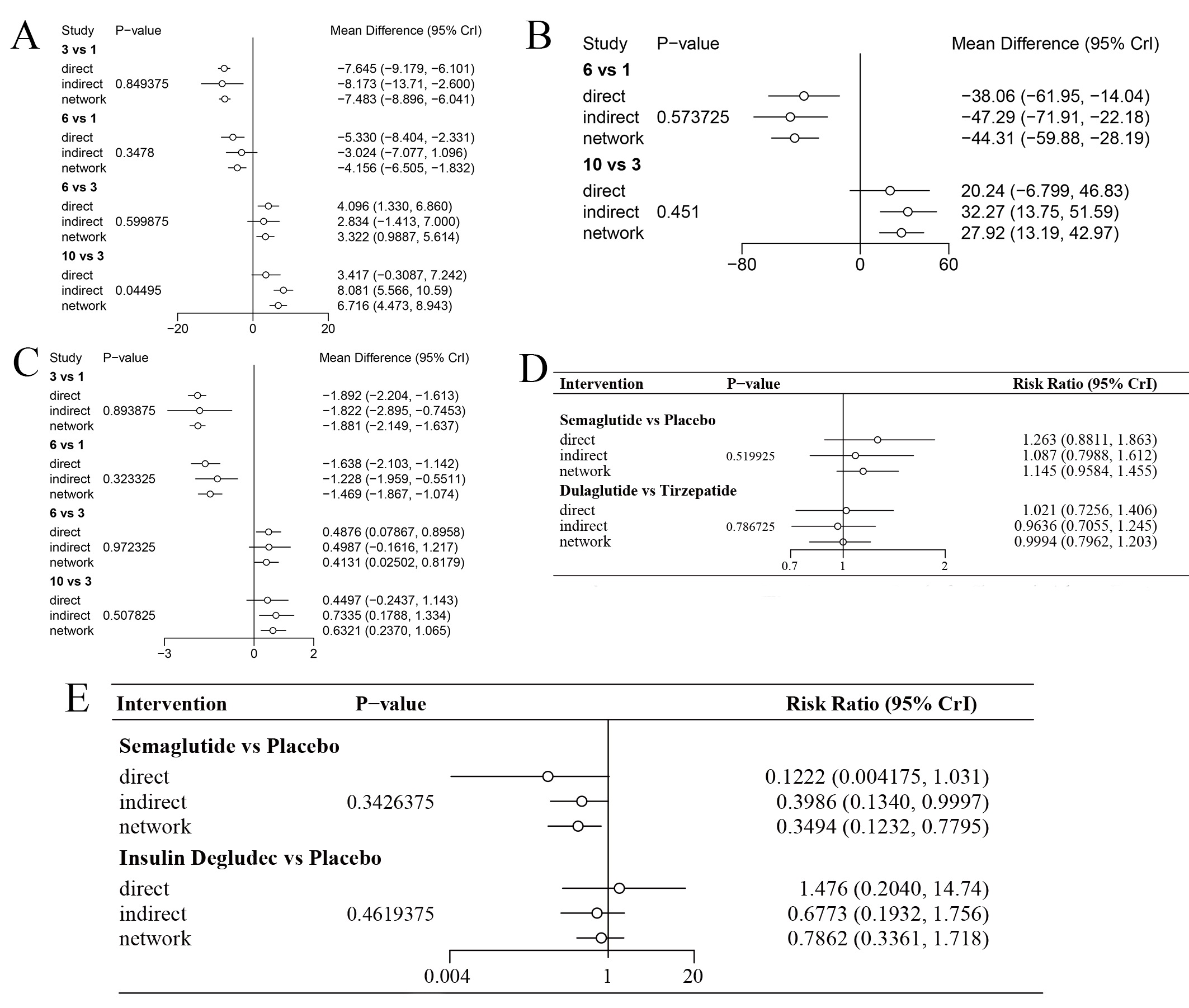

Supplement: Supplementary file 2 — Figure S1 Inconsistency Test Results for Weight Change, FBG, HbA1c, AEs, and SAEs Across Incretin-Based Therapies and Comparators in T2DM. Supplementary file2 (TIF 428 KB) [file 592_2025_2534_MOESM2_ESM.tif]

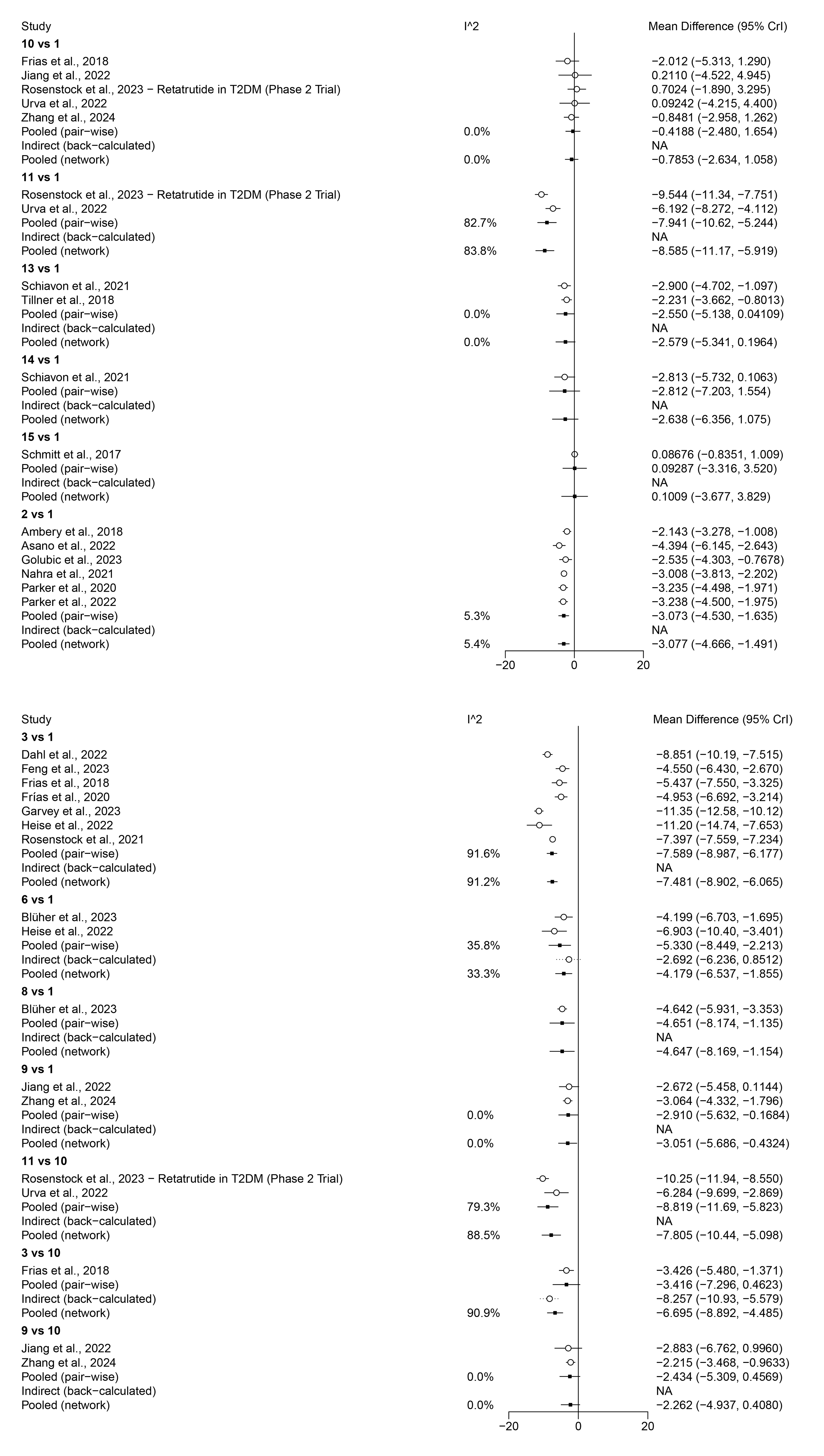

Supplement: Supplementary file 3 — Figure S2 Heterogeneity Test Results for Changes in Weight. Supplementary file3 (TIF 1442 KB) [file 592_2025_2534_MOESM3_ESM.tif]

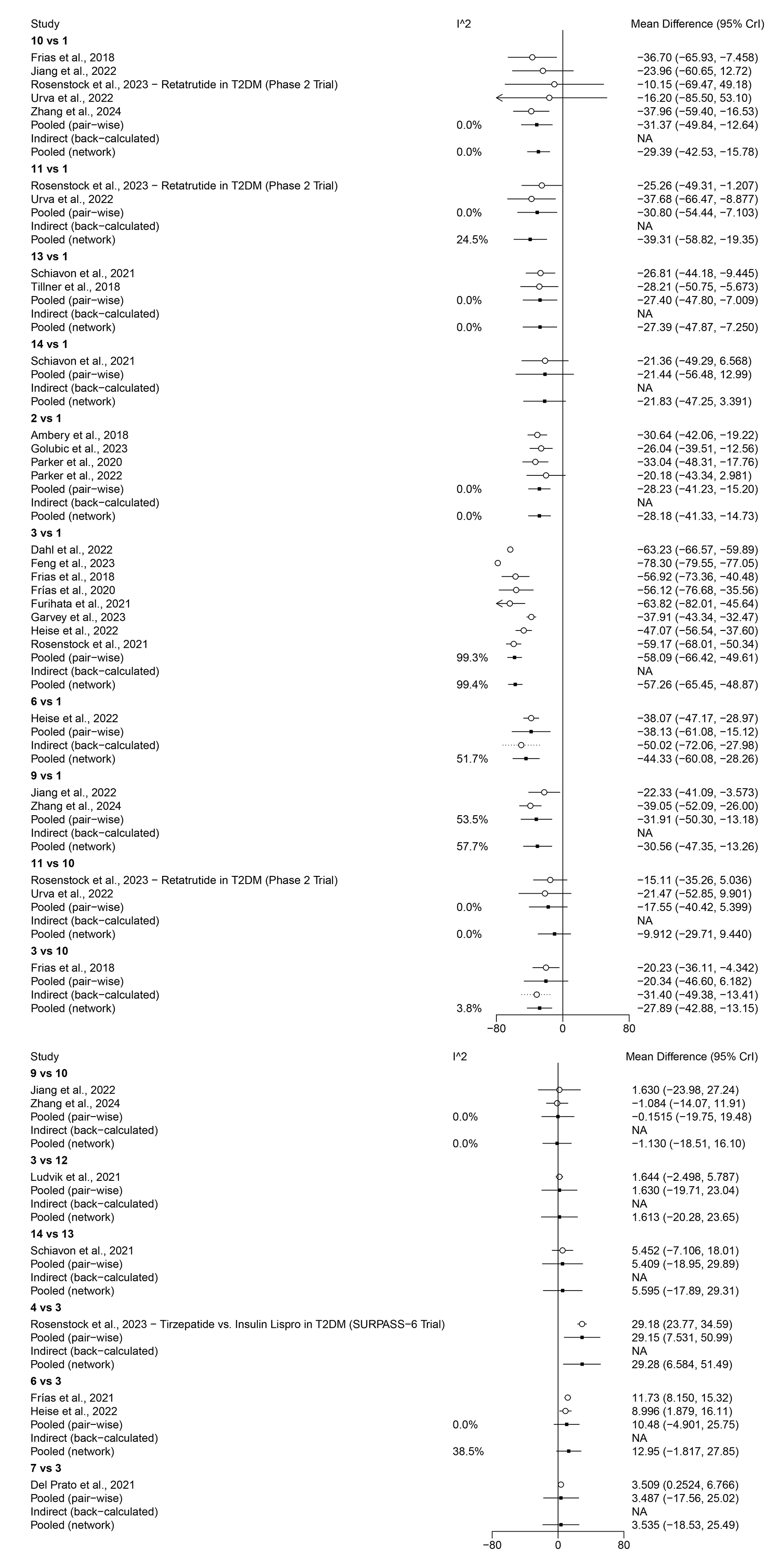

Supplement: Supplementary file 4 — Figure S3 Heterogeneity Test Results for Changes in Fasting Blood Glucose. Supplementary file4 (TIF 954 KB) [file 592_2025_2534_MOESM4_ESM.tif]

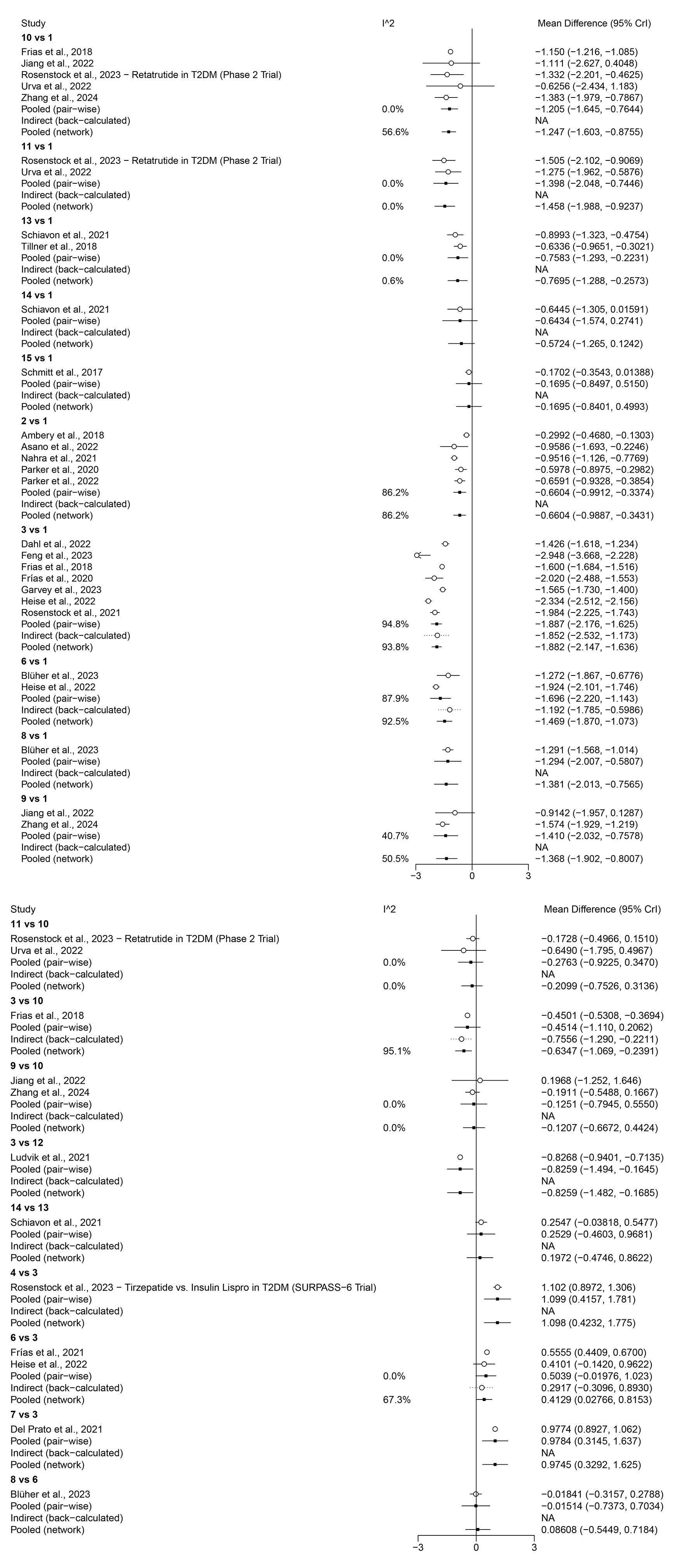

Supplement: Supplementary file 5 — Figure S4 Heterogeneity Test Results for Changes in HbA1c. Supplementary file5 (TIF 1101 KB) [file 592_2025_2534_MOESM5_ESM.tif]

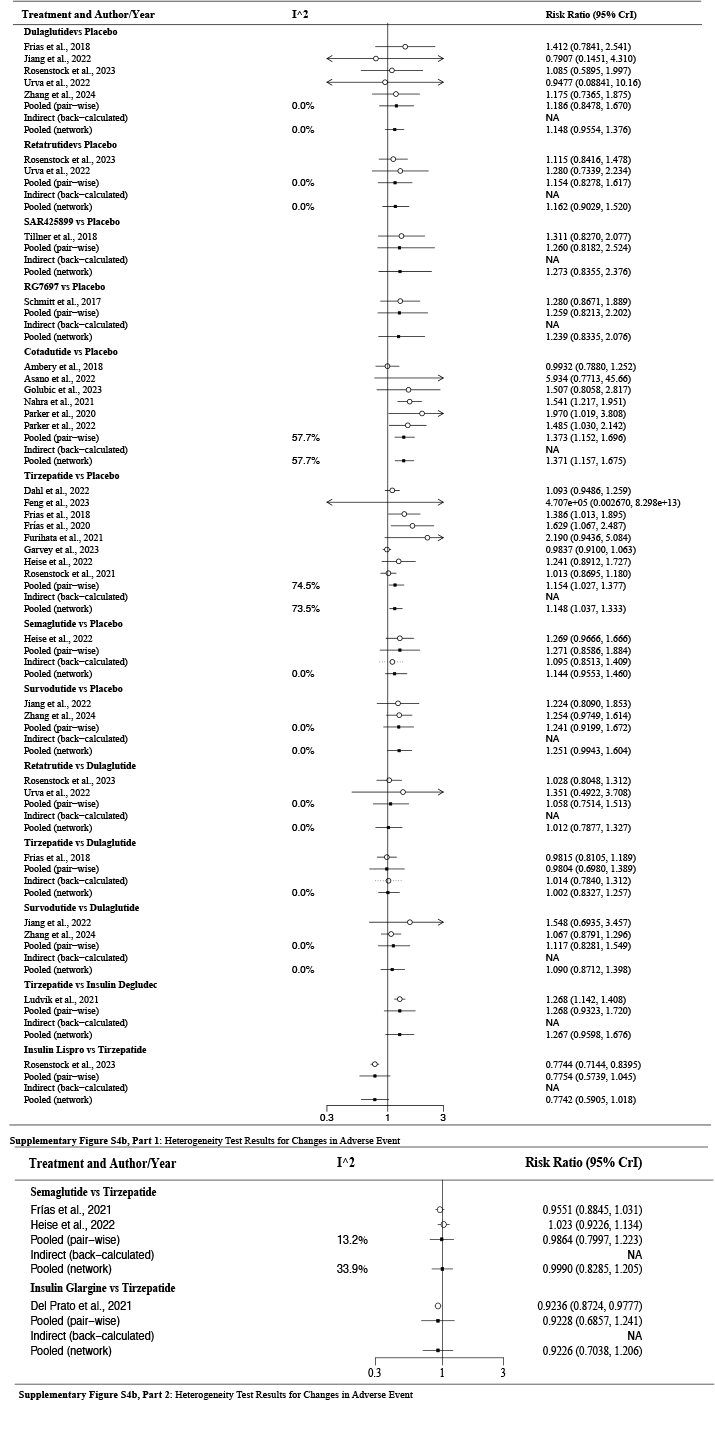

Supplement: Supplementary file 6 — Figure S5 Heterogeneity Test Results for Changes in Adverse Event. Supplementary file6 (TIF 167 KB) [file 592_2025_2534_MOESM6_ESM.tif]

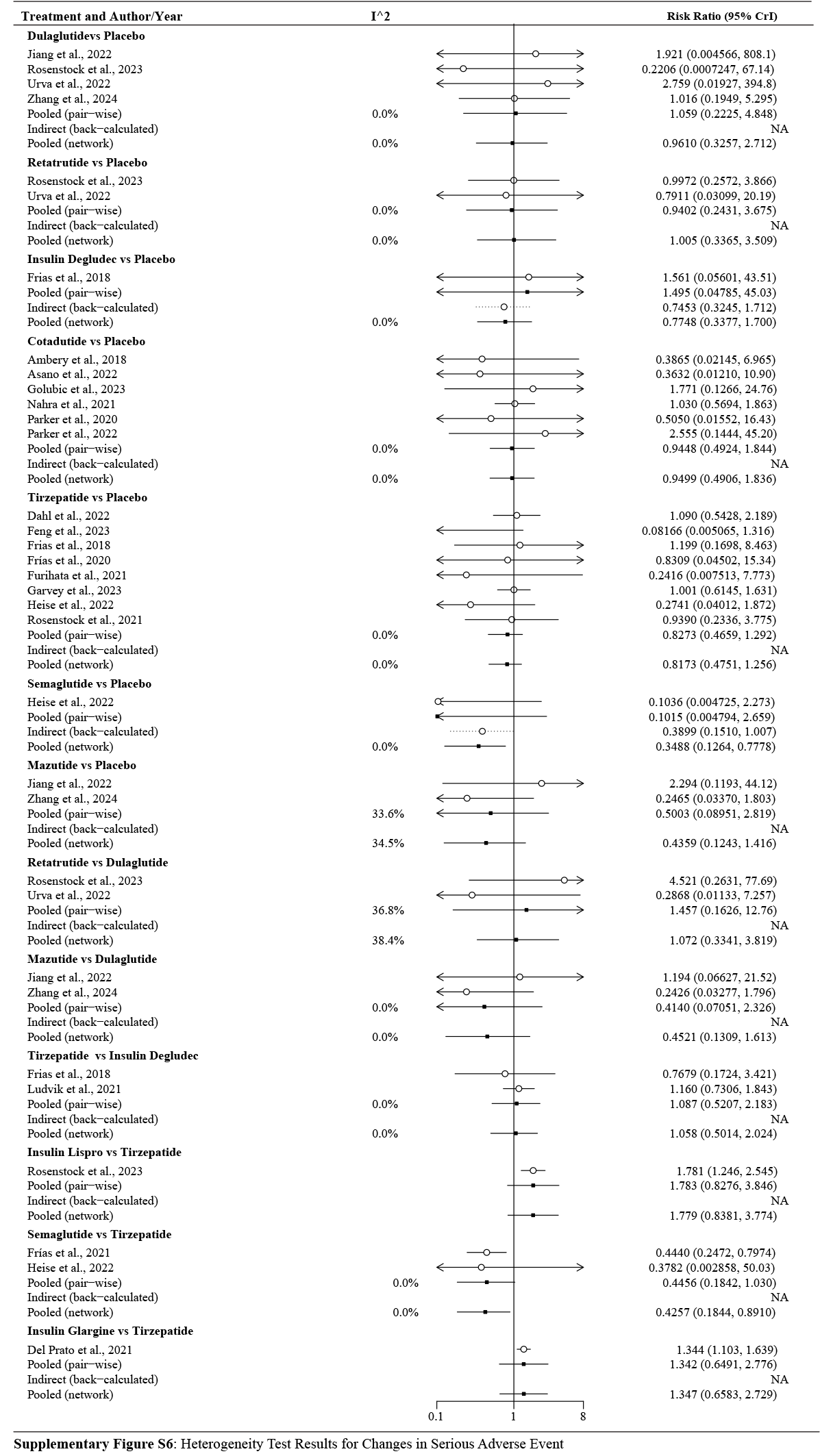

Supplement: Supplementary file 7 — Figure S6 Heterogeneity Test Results for Changes in Serious Adverse Event. Supplementary file7 (TIF 305 KB) [file 592_2025_2534_MOESM7_ESM.tif]

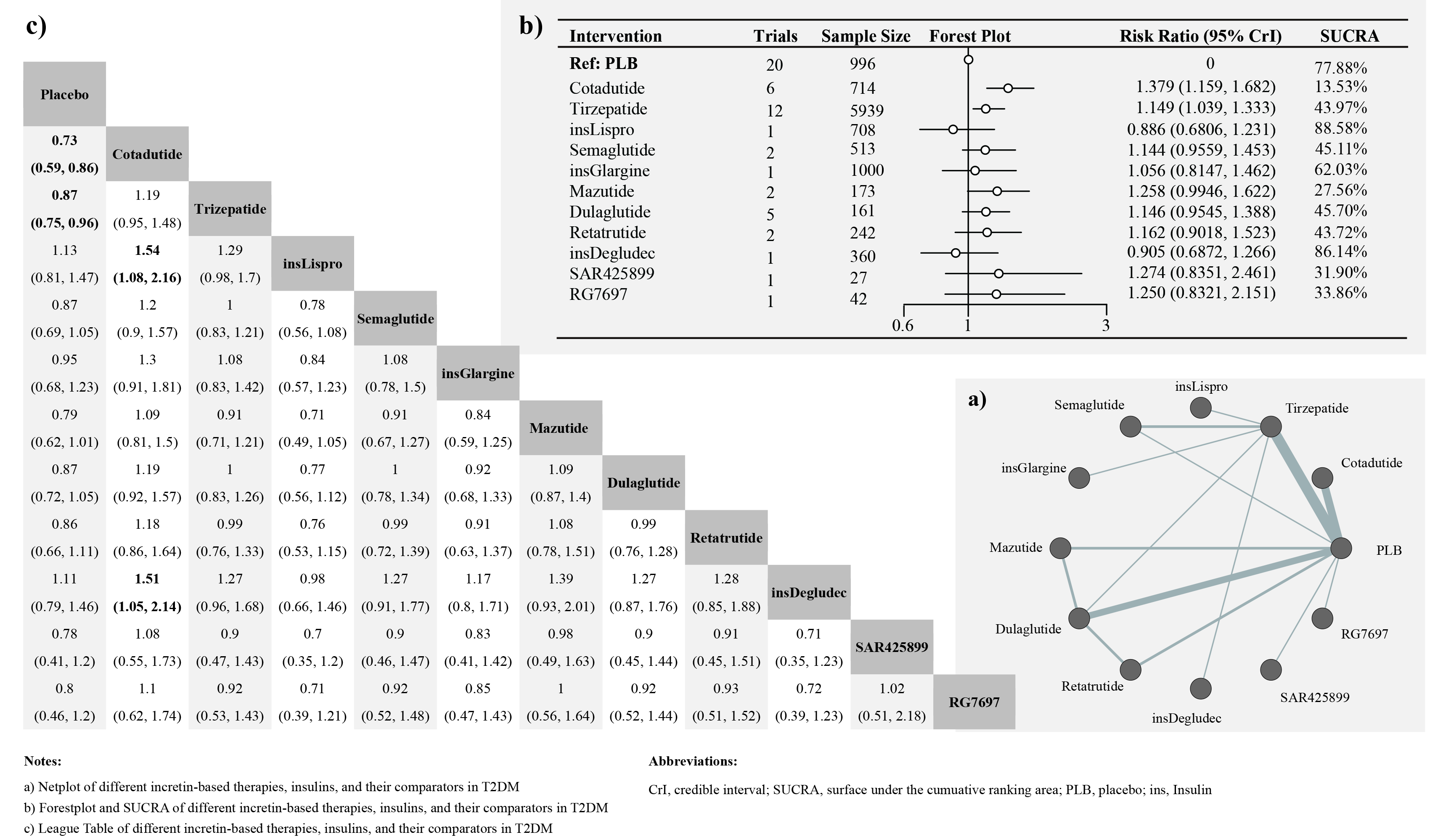

Supplement: Supplementary file 8 — Figure S7 Netplot, Forest Plot, and League Table of Adverse Events Risk Across Incretin-Based Therapies, Insulins, and Their Comparators in T2DM. Supplementary file8 (TIF 767 KB) [file 592_2025_2534_MOESM8_ESM.tif]

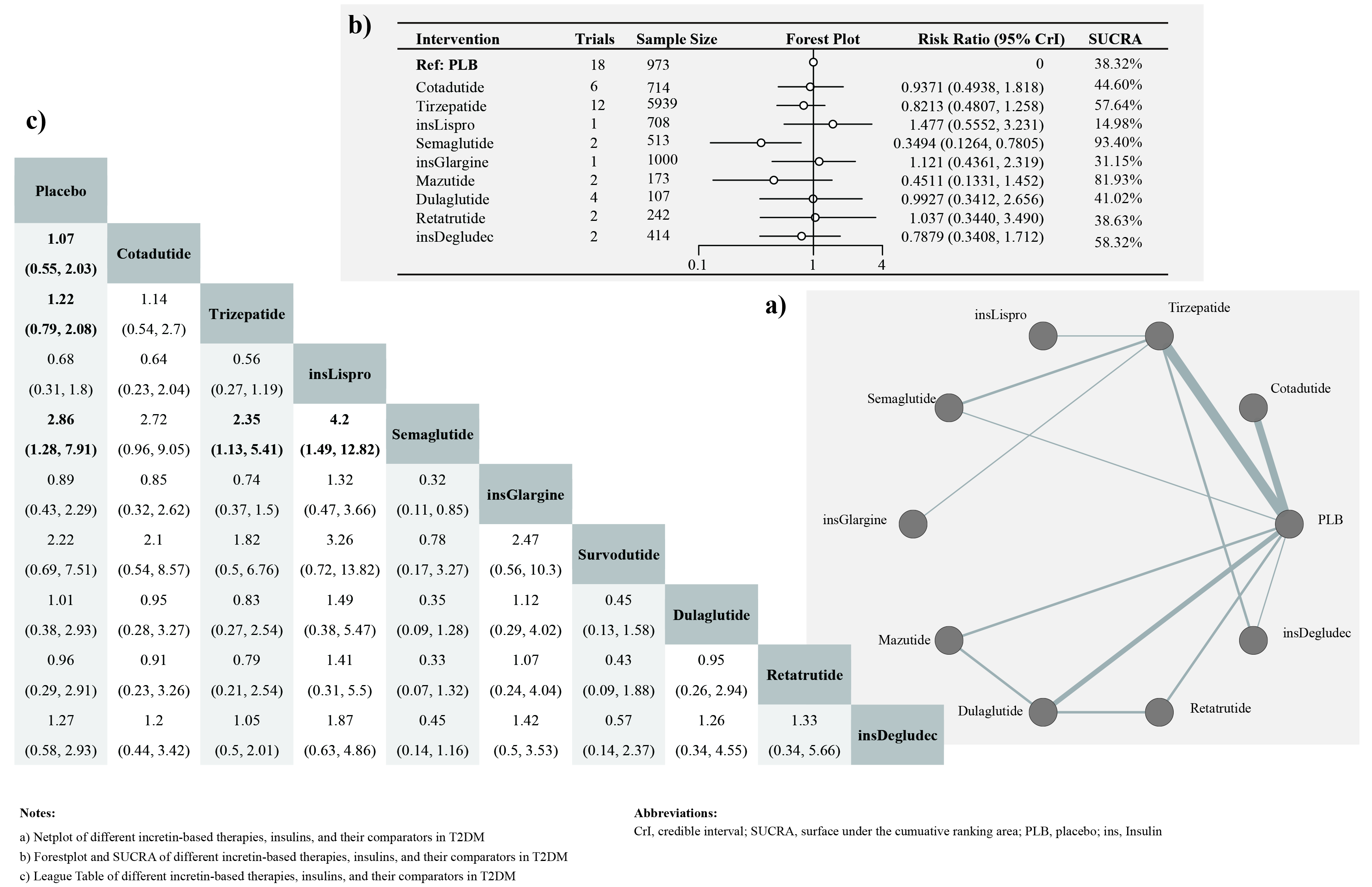

Supplement: Supplementary file 9 — Figure S8 Netplot, Forest Plot, and League Table of Serious Adverse Events Risk Across Incretin-Based Therapies, Insulins, and Their Comparators in T2DM. Supplementary file9 (TIF 797 KB) [file 592_2025_2534_MOESM9_ESM.tif]

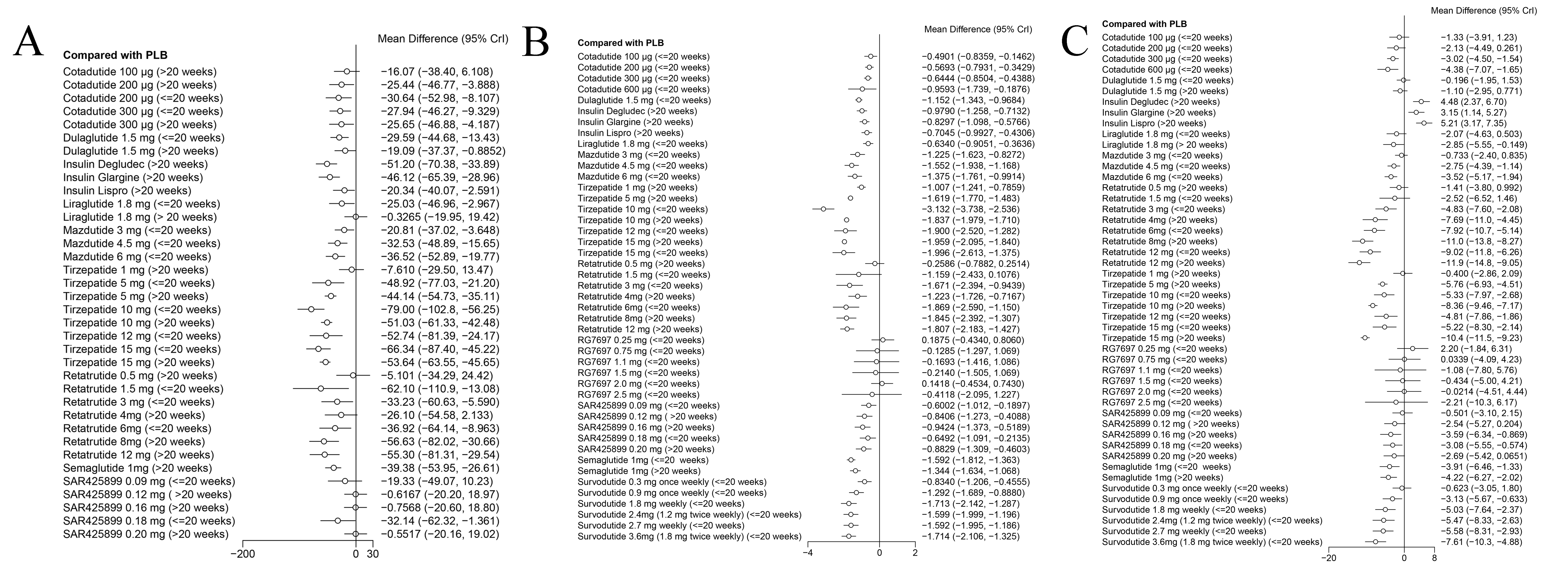

Supplement: Supplementary file 13 — Figure S12 Forest Plot for Impact of Dosage and Treatment Duration on FBG, HbA1C, Weight. Supplementary file13 (TIF 4485 KB) [file 592_2025_2534_MOESM13_ESM.tif]

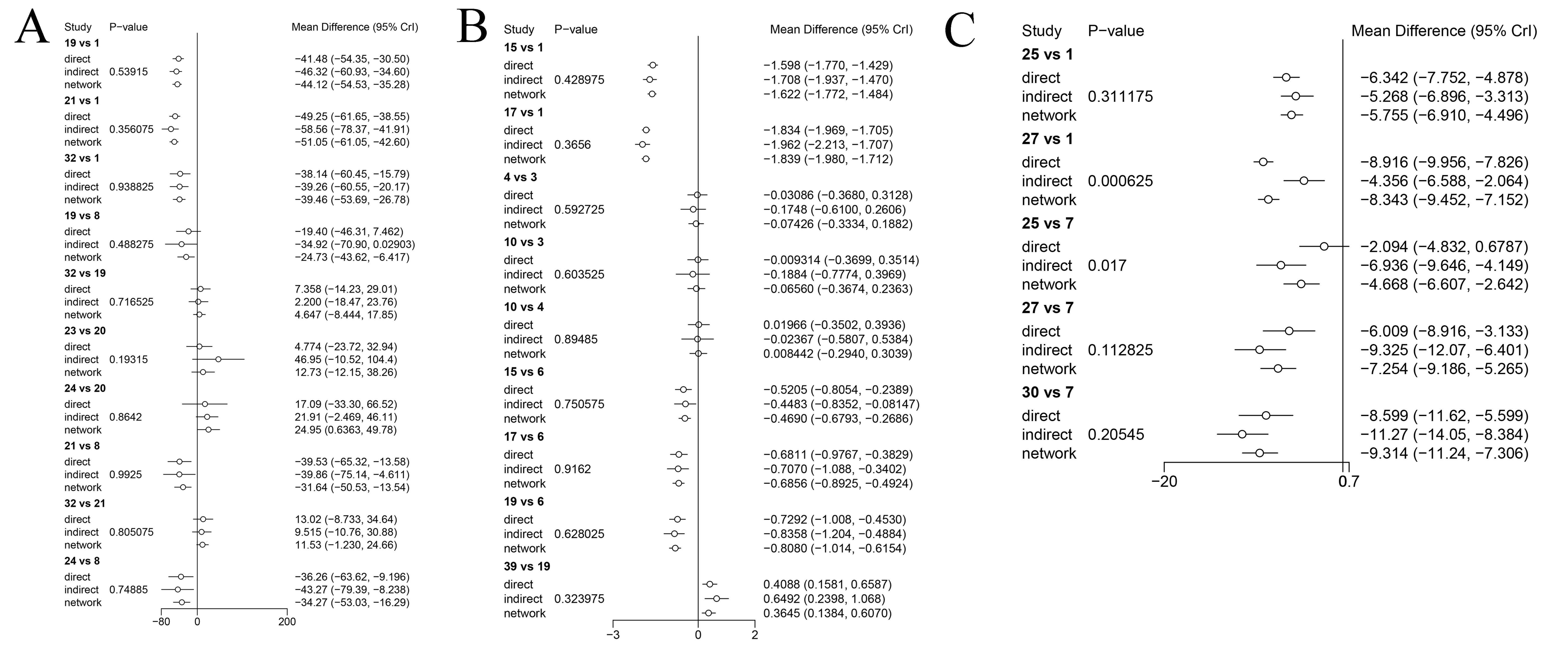

Supplement: Supplementary file 14 — Figure S13 Inconsistency Tests Results for Dosage and Treatment Duration Change on FBG, HbA1C, Weight. Supplementary file14 (TIF 2506 KB) [file 592_2025_2534_MOESM14_ESM.tif]
