# Supplementary material for: Beyond GLP-1: efficacy and safety of dual and triple incretin agonists in personalized type 2 diabetes care—a systematic review and network meta-analysis
Source: Acta Diabetol. 2025 Jun 5;62(9):1359–70. doi: 10.1007/s00592-025-02534-y (PMC12433336; doi:10.1007/s00592-025-02534-y)
Supplement: Supplementary file 10 — Figure S9 Impact of Dosage and Treatment Duration on Weight Change. Supplementary file10 (PDF 119 KB) [file 592_2025_2534_MOESM10_ESM.pdf]

Figure S9. Impact of Dosage and Treatment Duration on Weight Change

Author (Year)	NCT	Intervention	Dosage	Frequency	Duration
Asano M et al. (2022)	NCT04208620	Cotadutide (600 µg)	Cotadutide was titrated from 50 µg, escalating weekly to 600 µg by Day 36, and maintained at 600 µg until Day 70.	Once daily	70 days
Asano M et al. (2022)	NCT04208620	PLB	Volume-matched placebo following the same dose-escalation schedule as Cotadutide groups	Once daily	70 days
Ambery P et al. (2018)	NCT02548585	Cotadutide 200 µg	Up to 200 µg	Once daily	41 days
Ambery P et al. (2018)		PLB	Matched volume	Once daily	41 days
Blüher M et al. (2023)	NCT04153929	PLB	Volume- and schedule-matched to active intervention doses (e.g., once or twice weekly)	As per active intervention	16 weeks
Blüher M et al. (2023)	NCT04153929	Semaglutide (1 mg)	Initiated at 0.25 mg (Weeks 1–4) → 0.5 mg (Weeks 5–8) → 1.0 mg (Weeks 9–16)	Once weekly	16 weeks
Blüher M et al. (2023)	NCT04153929	Survodutide 0.3 mg weekly	0.3 mg maintained throughout the study period	Once weekly	16 weeks
Blüher M et al. (2023)	NCT04153929	Survodutide 0.9 mg weekly	0.3 mg (Weeks 1–4) → 0.6 mg (Weeks 5–8) → 0.9 mg (Weeks 9–16)	Once weekly	16 weeks
Blüher M et al. (2023)	NCT04153929	Survodutide 1.2 mg twice weekly (2.4mg)	0.3 mg (Weeks 1–4) → 0.6 mg (Weeks 5–8) → 1.2 mg (Weeks 9–16)	Twice weekly	16 weeks
Blüher M et al. (2023)	NCT04153929	Survodutide 1.8 mg twice weekly (3.6mg)	0.3 mg (Weeks 1–4) → 0.6 mg (Weeks 5–8) → 1.2 mg (Weeks 9–10) → 1.8 mg (Weeks 11–16)	Twice weekly	16 weeks
Blüher M et al. (2023)	NCT04153929	Survodutide 1.8 mg weekly	0.3 mg (Weeks 1–4) → 0.6 mg (Weeks 5–8) → 1.2 mg (Weeks 9–12) → 1.8 mg (Weeks 13–16)	Once weekly	16 weeks
Blüher M et al. (2023)	NCT04153929	Survodutide 2.7 mg weekly	0.3 mg (Weeks 1–4) → 0.6 mg (Weeks 5–8) → 1.2 mg (Weeks 9–12) → 2.4 mg (Weeks 13–14) → 2.7 mg (Weeks 15–16)	Once weekly	16 weeks
Dahl D et al. (2022)	NCT04039503	PLB	Volume-matched to Tirzepatide doses	Once weekly	40 weeks
Dahl D et al. (2022)	NCT04039503	Tirzepatide (10 mg)	Starting dose: 2.5 mg (Weeks 1–4) → 5 mg (Weeks 5–8) → Maintained at 10 mg from Week 9 onward	Once weekly	40 weeks
Dahl D et al. (2022)	NCT04039503	Tirzepatide (15 mg)	Starting dose: 2.5 mg (Weeks 1–4) → 5 mg (Weeks 5–8) → 10 mg (Weeks 9–12) → Maintained at 15 mg from Week 13 onward	Once weekly	40 weeks

Dahl D et al. (2022)	NCT04039503	Tirzepatide (5 mg)	Starting dose: 2.5 mg (Weeks 1–4) → Maintained at 5 mg from Week 5 onward	Once weekly	40 weeks
Del Prato S et al. (2021)	NCT03730662	Insulin Glargine (Basaglar)	Titrated starting at 10 U/day → Adjusted to target fasting glucose <100 mg/dL based on median of three blood glucose readings	Once daily	52 weeks
Del Prato S et al. (2021)	NCT03730662	Tirzepatide (10 mg)	Starting dose: 2.5 mg (Weeks 1–4) → 5 mg (Weeks 5–8) → Maintained at 10 mg (Week 9 onward)	Once weekly	52 weeks
Del Prato S et al. (2021)	NCT03730662	Tirzepatide (15 mg)	Starting dose: 2.5 mg (Weeks 1–4) → 5 mg (Weeks 5–8) → 10 mg (Weeks 9–12) → Maintained at 15 mg (Week 13 onward)	Once weekly	52 weeks
Del Prato S et al. (2021)	NCT03730662	Tirzepatide (5 mg)	Starting dose: 2.5 mg (Weeks 1–4) → Maintained at 5 mg (Week 5 onward)	Once weekly	52 weeks
Feng P et al. (2023)	NCT04235959	PLB	Volume- and frequency-matched placebo to Tirzepatide dosing regimens	Once weekly	16–24 weeks
Feng P et al. (2023)	NCT04235959	Tirzepatide: Cohort 1 (2.5–10.0 mg)	2.5 mg (Weeks 1–4) → Escalated by 2.5 mg every 4 weeks to 10.0 mg (Week 16)	Once weekly	16 weeks
Feng P et al. (2023)	NCT04235959	Tirzepatide: Cohort 2 (2.5–15.0 mg)	2.5 mg (Weeks 1–4) → Escalated by 2.5 mg every 4 weeks to 15.0 mg (Week 24)	Once weekly	24 weeks
Frías et al. (2021)	NCT03987919	Semaglutide (1 mg)	0.25 mg (Weeks 1–4) → 0.5 mg (Weeks 5–8) → Maintained at 1 mg (Week 9 onward)	Once weekly	40 weeks
Frías et al. (2021)	NCT03987919	Tirzepatide (10 mg)	2.5 mg (Weeks 1–4) → 5 mg (Weeks 5–8) → Maintained at 10 mg (Week 9 onward)	Once weekly	40 weeks
Frías et al. (2021)	NCT03987919	Tirzepatide (15 mg)	2.5 mg (Weeks 1–4) → 5 mg (Weeks 5–8) → 10 mg (Weeks 9–12) → Maintained at 15 mg (Week 13 onward)	Once weekly	40 weeks
Frías et al. (2021)	NCT03987919	Tirzepatide (5 mg)	2.5 mg (Weeks 1–4) → Maintained at 5 mg (Week 5 onward)	Once weekly	40 weeks
Frias JP et al. (2018)	NCT03131687	Dulaglutide (1.5 mg)	Fixed dose: 1.5 mg	Once weekly	26 weeks
Frias JP et al. (2018)	NCT03131687	PLB	Volume- and frequency-matched placebo for each active treatment arm	Once weekly	26 weeks
Frias JP et al. (2018)	NCT03131687	Tirzepatide (1 mg)	Fixed dose: 1 mg	Once weekly	26 weeks
Frias JP et al. (2018)	NCT03131687	Tirzepatide (10 mg)	Starting dose: 5 mg (Weeks 1–2) → Escalated to 10 mg (Weeks 3–26)	Once weekly	26 weeks

Frias JP et al. (2018)	NCT03131687	Tirzepatide (15 mg)	Starting dose: 5 mg (Weeks 1–2) → Escalated to 10 mg (Weeks 3–6) → 15 mg (Weeks 7–26)	Once weekly	26 weeks
Frias JP et al. (2018)	NCT03131687	Tirzepatide (5 mg)	Fixed dose: 5 mg	Once weekly	26 weeks
Frías JP et al. (2020)	NCT03311724	PLB	Volume-matched placebo following the same dose schedules as the Tirzepatide groups	Once weekly	12 weeks
Frías JP et al. (2020)	NCT03311724	Tirzepatide 12 mg	4 mg QW (Weeks 0–3) → 8 mg QW (Weeks 4–7) → 12 mg QW (Weeks 8–11)	Once weekly	12 weeks
Frías JP et al. (2020)	NCT03311724	Tirzepatide 15 mg (Accelerated Escalation)	2.5 mg (Weeks 0–1) → 5 mg (Weeks 2–3) → 10 mg (Weeks 4–7) → 15 mg (Weeks 8–11)	Once weekly	12 weeks
Frías JP et al. (2020)	NCT03311724	Tirzepatide 15 mg (Gradual Escalation)	2.5 mg (Weeks 0–3) → 7.5 mg (Weeks 4–7) → 15 mg (Weeks 8–11)	Once weekly	12 weeks
Furihata K et al. (2021)	NCT03322631	PLB	Volume- and frequency-matched placebo	Once weekly	8 weeks
Furihata K et al. (2021)	NCT03322631	Tirzepatide (10 mg)	2.5 mg (Weeks 1–2) → 5 mg (Weeks 3–4) → 10 mg (Weeks 5–8)	Once weekly	8 weeks
Furihata K et al. (2021)	NCT03322631	Tirzepatide (15 mg)	5 mg (Weeks 1–2) → 10 mg (Weeks 3–6) → 15 mg (Weeks 7–8)	Once weekly	8 weeks
Furihata K et al. (2021)	NCT03322631	Tirzepatide (5 mg)	Fixed dose: 5 mg (Weeks 1–8)	Once weekly	8 weeks
Garvey WT et al. (2023)	NCT04657003	PLB	Volume-matched placebo injections to mimic the Tirzepatide dose-escalation schedule	Once weekly	72 weeks
Garvey WT et al. (2023)	NCT04657003	Tirzepatide (10 mg)	Starting dose: 2.5 mg (Weeks 1–4) → 5 mg (Weeks 5–8) → 7.5 mg (Weeks 9–12) → Maintained at 10 mg (Week 13 onward)	Once weekly	72 weeks
Garvey WT et al. (2023)	NCT04657003	Tirzepatide (15 mg)	Starting dose: 2.5 mg (Weeks 1–4) → 5 mg (Weeks 5–8) → 7.5 mg (Weeks 9–12) → 10 mg (Weeks 13–16) → Maintained at 15 mg (Week 17 onward)	Once weekly	72 weeks
Golubic R et al. (2023)	NCT03596177	Cotadutide (300 μg)	Initiated at 100 μg/day (Days 1–4) → 200 μg/day (Days 5–8) → 300 μg/day (Days 9–42)	Once daily	Day 59
Golubic R et al. (2023)	NCT03596177	PLB	Volume-matched to Cotadutide doses	Once daily	Day 59
Jiang H et al. (2022)	NCT04466904	Dulaglutide (1.5 mg)	Fixed dose: 1.5 mg	Once weekly	12 weeks

Jiang H et al. (2022)	NCT04466904	Mazutide (3.0 mg)	Dose escalation: 1.0 mg (Weeks 1–4) → 2.0 mg (Weeks 5–8) → 3.0 mg (Weeks 9–12)	Once weekly	12 weeks
Jiang H et al. (2022)	NCT04466904	Mazutide (4.5 mg)	Dose escalation: 1.5 mg (Weeks 1–4) → 3.0 mg (Weeks 5–8) → 4.5 mg (Weeks 9–12)	Once weekly	12 weeks
Jiang H et al. (2022)	NCT04466904	Mazutide (6.0 mg)	Dose escalation: 2.0 mg (Weeks 1–4) → 4.0 mg (Weeks 5–8) → 6.0 mg (Weeks 9–12)	Once weekly	12 weeks
Jiang H et al. (2022)	NCT04466904	PLB	Volume-matched placebo following the same dose-escalation schedules as IBI362 groups	Once weekly	12 weeks
Ludvik B et al. (2021)	NCT03882970	Insulin Degludec	Initial dose: 10 units/day → Weekly titration to fasting blood glucose target (<90 mg/dL)	Once weekly	52 weeks
Ludvik B et al. (2021)	NCT03882970	Tirzepatide: 10 mg	Starting dose: 2.5 mg (4 weeks) → Escalated to 5 mg (4 weeks) → 10 mg (Week 9 onward)	Once weekly	52 weeks
Ludvik B et al. (2021)	NCT03882970	Tirzepatide: 15 mg	Starting dose: 2.5 mg (4 weeks) → Escalated to 5 mg (4 weeks) → 10 mg (4 weeks) → 15 mg (Week 13 onward)	Once weekly	52 weeks
Ludvik B et al. (2021)	NCT03882970	Tirzepatide: 5 mg	Starting dose: 2.5 mg (4 weeks) → Escalated to 5 mg (Week 5 onward)	Once weekly	52 weeks
Nahra R et al. (2021)	NCT03235050	Cotadutide 100 µg	100 µg once daily	Once daily	14 weeks
Nahra R et al. (2021)	NCT03235050	Cotadutide 200 µg	100 µg initial dose → Titrated to 200 µg/day	Once daily	14 weeks
Nahra R et al. (2021)	NCT03235050	Cotadutide 300 µg	100 µg initial dose → Titrated to 300 µg/day	Once daily	14 weeks
Nahra R et al. (2021)	NCT03235050	Liraglutide 1.8 mg	0.6 mg starting dose, up titrated weekly by 0.6 mg until 1.8 mg/day	Once daily	14 weeks
Nahra R et al. (2021)	NCT03235050	PLB	Volume- and frequency-matched placebo for Cotadutide dosing	Once daily	14 weeks
Parker VE et al. (2020)	NCT03244800	Cotadutide (Cohort 1 -300 µg maintained for 28 days (Days 22–49)	50 µg/day (Days 1–7) → 100 µg/day (Days 8–14) → 200 µg/day (Days 15–21) → 300 µg/day (Days 22–49)	Once daily	49 days
Parker VE et al. (2020)	NCT03244800	Cotadutide (Cohort 2 -300 µg maintained for 7 days Days 43–49)	50 µg/day (Days 1–14) → 100 µg/day (Days 15–28) → 200 µg/day (Days 29–42) → 300 µg/day (Days 43–49)	Once daily	49 days
Parker VE et al. (2020)	NCT03244800	PLB	Volume-matched placebo following the same dose-escalation schedules as Cotadutide group	Once daily	49 days
Parker VER et al. (2022)	NCT03550378	Cotadutide (300 µg)	50 µg (Days 1–4) → Escalated to 100 µg (Days 5–11) → 200 µg (Days 12–18) → 300 µg (Days 19–32)	Once daily	32 days

Parker VER et al. (2022)		PLB	Volume- and frequency-matched placebo injections	Once daily	32 days
Rosenstock J et al. (2021)	NCT03954834	PLB	Volume-matched to Tirzepatide doses	Once weekly	40 weeks
Rosenstock J et al. (2021)	NCT03954834	Tirzepatide (10 mg)	2.5 mg (Weeks 1–4) → 5 mg (Weeks 5–8) → Maintained at 10 mg (Week 9 onward)	Once weekly	40 weeks
Rosenstock J et al. (2021)	NCT03954834	Tirzepatide (15 mg)	2.5 mg (Weeks 1–4) → 5 mg (Weeks 5–8) → 10 mg (Weeks 9–12) → Maintained at 15 mg (Week 13 onward)	Once weekly	40 weeks
Rosenstock J et al. (2021)	NCT03954834	Tirzepatide (5 mg)	2.5 mg (Weeks 1–4) → Maintained at 5 mg (Week 5 onward)	Once weekly	40 weeks
Rosenstock J et al. (2023) - Dulaglutide	NCT04867785	Dulaglutide (1.5 mg)	Fixed dose: 1.5 mg	Once weekly	36 weeks
Rosenstock J et al. (2023) - Dulaglutide	NCT04867785	PLB	Volume-matched placebo following the same dose-escalation schedules as Retatrutide groups	Once weekly	36 weeks
Rosenstock J et al. (2023) - Dulaglutide	NCT04867785	Retatrutide (0.5 mg)	0.5 mg group: Fixed	Once weekly	36 weeks
Rosenstock J et al. (2023) - Dulaglutide	NCT04867785	Retatrutide (12 mg Escalation Group)	2 mg (Weeks 1–4) → 4 mg (Weeks 5–8) → 8 mg (Weeks 9–12) → 12 mg (Weeks 13–36)	Once weekly	36 weeks
Rosenstock J et al. (2023) - Dulaglutide	NCT04867785	Retatrutide (4 mg Escalation Group)	2 mg (Weeks 1–4) → 4 mg (Weeks 5–36)	Once weekly	36 weeks
Rosenstock J et al. (2023) - Dulaglutide	NCT04867785	Retatrutide (4 mg No Escalation Group)	Fixed dose of 4 mg	Once weekly	36 weeks
Rosenstock J et al. (2023) - Dulaglutide	NCT04867785	Retatrutide (8 mg Fast Escalation)	4 mg (Weeks 1–4) → 8 mg (Weeks 5–36)	Once weekly	36 weeks
Rosenstock J et al. (2023) - Dulaglutide	NCT04867785	Retatrutide (8 mg Slow Escalation)	2 mg (Weeks 1–4) → 4 mg (Weeks 5–8) → 8 mg (Weeks 9–36)	Once weekly	36 weeks
Rosenstock J et al. (2023) - Insulin Lispro	NCT04537923	Insulin Lispro	Initial dose: 4 IU before each meal, adjusted based on fasting/pre-prandial glucose levels	Thrice daily	52 weeks
Rosenstock J et al. (2023) - Insulin Lispro	NCT04537923	Tirzepatide (10 mg)	2.5 mg (Weeks 1–4) → 5 mg (Weeks 5–8) → Escalated to 10 mg from Week 9 onward	Once weekly	52 weeks

Rosenstock J et al. (2023) - Insulin Lispro	NCT04537923	Tirzepatide (15 mg)	2.5 mg (Weeks 1–4) → 5 mg (Weeks 5–8) → 10 mg (Weeks 9–12) → Escalated to 15 mg from Week 13 onward	Once weekly	52 weeks
Rosenstock J et al. (2023) - Insulin Lispro	NCT04537923	Tirzepatide (5 mg)	2.5 mg (Weeks 1–4) → Maintained at 5 mg from Week 5 onward	Once weekly	52 weeks
Schiavon M et al. (2021)	NCT02973321	Liraglutide (1.80 mg)	Fixed dose: 1.80 mg/day	Once daily	26 weeks
Schiavon M et al. (2021)	NCT02973321	PLB	Volume- and frequency-matched placebo for each active treatment arm	Once daily	26 weeks
Schiavon M et al. (2021)	NCT02973321	SAR425899 (0.12 mg)	Fixed dose: 0.12 mg/day	Once daily	26 weeks
Schiavon M et al. (2021)	NCT02973321	SAR425899 (0.16 mg)	Fixed dose: 0.16 mg/day	Once daily	26 weeks
Schiavon M et al. (2021)	NCT02973321	SAR425899 (0.20 mg)	Fixed dose: 0.20 mg/day	Once daily	26 weeks
Schmitt C et al. (2017)	NCT01789788	PLB	Volume- and frequency-matched placebo for each active treatment arm	Once daily	14 days
Schmitt C et al. (2017)	NCT01789788	RG7697 (0.25 mg)	Fixed dose: 0.25 mg/day	Once daily	14 days
Schmitt C et al. (2017)	NCT01789788	RG7697 (0.75 mg)	Fixed dose: 0.75 mg/day	Once daily	14 days
Schmitt C et al. (2017)	NCT01789788	RG7697 (1.1 mg)	Fixed dose: 1.1 mg/day	Once daily	14 days
Schmitt C et al. (2017)	NCT01789788	RG7697 (1.5 mg)	Fixed dose: 1.5 mg/day	Once daily	14 days
Schmitt C et al. (2017)	NCT01789788	RG7697 (2.0 mg)	Fixed dose: 2.0 mg/day	Once daily	14 days
Schmitt C et al. (2017)	NCT01789788	RG7697 (2.5 mg)	Fixed dose: 2.5 mg/day	Once daily	14 days
Tillner J et al. (2018)	NCT02411825	PLB	Volume-matched placebo following the same dose-escalation schedules as SAR425899 groups	Once daily	28 days
Tillner J et al. (2018)	NCT02411825	SAR425899 (0.09mg)	0.03 mg (Days 1–7) → 0.06 mg (Days 8–14) → 0.09 mg (Days 15–21)	Once daily	28 days
Tillner J et al. (2018)	NCT02411825	SAR425899 (0.18mg)	0.06 mg (Days 1–7) → 0.12 mg (Days 8–14) → 0.18 mg (Days 15–21)	Once daily	28 days
Urva S et al. (2022)	NCT04143802	Dulaglutide (1.5 mg)	Fixed dose: 1.5 mg	Once weekly	12 weeks
Urva S et al. (2022)	NCT04143802	PLB	Volume-matched placebo following the same dose-escalation schedules as Retatrutide groups	Once weekly	12 weeks
Urva S et al. (2022)	NCT04143802	Retatrutide: 0.5 mg (Fixed Dose)	0.5 mg weekly for 12 weeks	Once weekly	12 weeks

Urva S et al. (2022)	NCT04143802	Retatrutide: 1.5 mg (Fixed Dose)	1.5 mg weekly for 12 weeks	Once weekly	12 weeks
Urva S et al. (2022)	NCT04143802	Retatrutide: 3 mg (Fixed Dose)	3 mg weekly for 12 weeks	Once weekly	12 weeks
Urva S et al. (2022)	NCT04143802	Retatrutide: 3/6 mg (Gradual Escalation)	3 mg (Weeks 1–4) → 6 mg (Weeks 5–12)	Once weekly	12 weeks
Urva S et al. (2022)	NCT04143802	Retatrutide: 3/6/9/12 mg (Extended Escalation)	3 mg (Weeks 1–2) → 6 mg (Weeks 3–4) → 9 mg (Weeks 5–8) → 12 mg (Weeks 9–12)	Once weekly	12 weeks
Zhang B et al. (2024)	NA	Dulaglutide (1.5 mg)	Fixed dose: 1.5 mg	Once weekly	20 weeks
Zhang B et al. (2024)	NA	Mazdutide 3 mg	1.5 mg (Weeks 1–4) → 3 mg (Weeks 5–20)	Once weekly	20 weeks
Zhang B et al. (2024)	NA	Mazdutide 4.5 mg	1.5 mg (Weeks 1–4) → 3 mg (Weeks 5–8) → 4.5 mg (Weeks 9–20)	Once weekly	20 weeks
Zhang B et al. (2024)	NA	Mazdutide 6 mg	2 mg (Weeks 1–4) → 4 mg (Weeks 5–8) → 6 mg (Weeks 9–20)	Once weekly	20 weeks
Zhang B et al. (2024)	NA	PLB	Volume-matched placebo mimicking the Mazdutide dose-escalation schedules	Once weekly	20 weeks
