# Supplementary material for: Beyond GLP-1: efficacy and safety of dual and triple incretin agonists in personalized type 2 diabetes care—a systematic review and network meta-analysis
Source: Acta Diabetol. 2025 Jun 5;62(9):1359–70. doi: 10.1007/s00592-025-02534-y (PMC12433336; doi:10.1007/s00592-025-02534-y)
Supplement: Supplementary file 11 — Figure S10 Impact of Dosage and Treatment Duration on FBG Reduction. Supplementary file11 (PDF 147 KB) [file 592_2025_2534_MOESM11_ESM.pdf]

**Figure S10. Impact of Dosage and Treatment Duration on FBG Reduction**

| | | | | | |
|---|---|---|---|---|---|
| Ambery P et al. (2018) | NCT02548585 | MEDI0382 (Cotadutide) | Up to 200 µg | Once daily | 41 days |
| Ambery P et al. (2018) | | PLB | Matched volume | Once daily | 41 days |
| Dahl D et al. (2022) | NCT04039503 | Tirzepatide (5 mg) | Starting dose: 2.5 mg (Weeks 1–4) → Maintained at 5 mg from Week 5 onward | Once weekly | 40 weeks |
| Dahl D et al. (2022) | NCT04039503 | Tirzepatide (10 mg) | Starting dose: 2.5 mg (Weeks 1–4) → 5 mg (Weeks 5–8) → Maintained at 10 mg from Week 9 onward | Once weekly | 40 weeks |
| Dahl D et al. (2022) | NCT04039503 | Tirzepatide (15 mg) | Starting dose: 2.5 mg (Weeks 1–4) → 5 mg (Weeks 5–8) → 10 mg (Weeks 9–12) → Maintained at 15 mg from Week 13 onward | Once weekly | 40 weeks |
| Dahl D et al. (2022) | NCT04039503 | PLB | Volume-matched to Tirzepatide doses | Once weekly | 40 weeks |
| Del Prato S et al. (2021) | NCT03730662 | Tirzepatide (5 mg) | Starting dose: 2.5 mg (Weeks 1–4) → Maintained at 5 mg (Week 5 onward) | Once weekly | 52 weeks |
| Del Prato S et al. (2021) | NCT03730662 | Tirzepatide (10 mg) | Starting dose: 2.5 mg (Weeks 1–4) → 5 mg (Weeks 5–8) → Maintained at 10 mg (Week 9 onward) | Once weekly | 52 weeks |
| Del Prato S et al. (2021) | NCT03730662 | Tirzepatide (15 mg) | Starting dose: 2.5 mg (Weeks 1–4) → 5 mg (Weeks 5–8) → 10 mg (Weeks 9–12) → Maintained at 15 mg (Week 13 onward) | Once weekly | 52 weeks |
| Del Prato S et al. (2021) | NCT03730662 | Insulin Glargine (Basaglar) | Titrated starting at 10 U/day → Adjusted to target fasting glucose <100 mg/dL based on median of three blood glucose readings | Once daily | 52 weeks |
| Feng P et al. (2023) | NCT04235959 | Tirzepatide: Cohort 1 (2.5–10.0 mg) | 2.5 mg (Weeks 1–4) → Escalated by 2.5 mg every 4 weeks to 10.0 mg (Week 16) | Once weekly | 16 weeks |
| Feng P et al. (2023) | NCT04235959 | Tirzepatide: Cohort 2 (2.5–15.0 mg) | 2.5 mg (Weeks 1–4) → Escalated by 2.5 mg every 4 weeks to 15.0 mg (Week 24) | Once weekly | 24 weeks |
| Feng P et al. (2023) | NCT04235959 | PLB | Volume- and frequency-matched placebo to Tirzepatide dosing regimens | Once weekly | 16–24 weeks |
| Frías et al. (2021) | NCT03987919 | Tirzepatide (5 mg) | 2.5 mg (Weeks 1–4) → Maintained at 5 mg (Week 5 onward) | Once weekly | 40 weeks |

| | | | | | |
|---|---|---|---|---|---|
| Frías et al. (2021) | NCT03987919 | Tirzepatide (10 mg) | 2.5 mg (Weeks 1–4) → 5 mg (Weeks 5–8) → Maintained at 10 mg (Week 9 onward) | Once weekly | 40 weeks |
| Frías et al. (2021) | NCT03987919 | Tirzepatide (15 mg) | 2.5 mg (Weeks 1–4) → 5 mg (Weeks 5–8) → 10 mg (Weeks 9–12) → Maintained at 15 mg (Week 13 onward) | Once weekly | 40 weeks |
| Frías et al. (2021) | NCT03987919 | Semaglutide (1 mg) | 0.25 mg (Weeks 1–4) → 0.5 mg (Weeks 5–8) → Maintained at 1 mg (Week 9 onward) | Once weekly | 40 weeks |
| Frias JP et al. (2018) | NCT03131687 | Tirzepatide (1 mg) | Fixed dose: 1 mg | Once weekly | 26 weeks |
| Frias JP et al. (2018) | NCT03131687 | Tirzepatide (5 mg) | Fixed dose: 5 mg | Once weekly | 26 weeks |
| Frias JP et al. (2018) | NCT03131687 | Tirzepatide (10 mg) | Starting dose: 5 mg (Weeks 1–2) → Escalated to 10 mg (Weeks 3–26) | Once weekly | 26 weeks |
| Frias JP et al. (2018) | NCT03131687 | Tirzepatide (15 mg) | Starting dose: 5 mg (Weeks 1–2) → Escalated to 10 mg (Weeks 3–6) → 15 mg (Weeks 7–26) | Once weekly | 26 weeks |
| Frias JP et al. (2018) | NCT03131687 | Dulaglutide (1.5 mg) | Fixed dose: 1.5 mg | Once weekly | 26 weeks |
| Frias JP et al. (2018) | NCT03131687 | PLB | Volume- and frequency-matched placebo for each active treatment arm | Once weekly | 26 weeks |
| Frías JP et al. (2020) | NCT03311724 | Tirzepatide 12 mg | 4 mg QW (Weeks 0–3) → 8 mg QW (Weeks 4–7) → 12 mg QW (Weeks 8–11) | Once weekly | 12 weeks |
| Frías JP et al. (2020) | NCT03311724 | Tirzepatide 15 mg (Gradual Escalation) | 2.5 mg (Weeks 0–3) → 7.5 mg (Weeks 4–7) → 15 mg (Weeks 8–11) | Once weekly | 12 weeks |
| Frías JP et al. (2020) | NCT03311724 | Tirzepatide 15 mg (Accelerated Escalation) | 2.5 mg (Weeks 0–1) → 5 mg (Weeks 2–3) → 10 mg (Weeks 4–7) → 15 mg (Weeks 8–11) | Once weekly | 12 weeks |
| Frías JP et al. (2020) | NCT03311724 | PLB | Volume-matched placebo following the same dose schedules as the Tirzepatide groups | Once weekly | 12 weeks |
| Furihata K et al. (2021) | NCT03322631 | Tirzepatide (5 mg) | Fixed dose: 5 mg (Weeks 1–8) | Once weekly | 8 weeks |
| Furihata K et al. (2021) | NCT03322631 | Tirzepatide (10 mg) | 2.5 mg (Weeks 1–2) → 5 mg (Weeks 3–4) → 10 mg (Weeks 5–8) | Once weekly | 8 weeks |
| Furihata K et al. (2021) | NCT03322631 | Tirzepatide (15 mg) | 5 mg (Weeks 1–2) → 10 mg (Weeks 3–6) → 15 mg (Weeks 7–8) | Once weekly | 8 weeks |
| Furihata K et al. (2021) | NCT03322631 | PLB | Volume- and frequency-matched placebo | Once weekly | 8 weeks |

| | | | | | |
|---|---|---|---|---|---|
| Garvey WT et al. (2023) | NCT04657003 | Tirzepatide (10 mg) | Starting dose: 2.5 mg (Weeks 1–4) → 5 mg (Weeks 5–8) → 7.5 mg (Weeks 9–12) → Maintained at 10 mg (Week 13 onward) | Once weekly | 72 weeks |
| Garvey WT et al. (2023) | NCT04657003 | Tirzepatide (15 mg) | Starting dose: 2.5 mg (Weeks 1–4) → 5 mg (Weeks 5–8) → 7.5 mg (Weeks 9–12) → 10 mg (Weeks 13–16) → Maintained at 15 mg (Week 17 onward) | Once weekly | 72 weeks |
| Garvey WT et al. (2023) | NCT04657003 | PLB | Volume-matched placebo injections to mimic the Tirzepatide dose-escalation schedule | Once weekly | 72 weeks |
| Heise T et al. (2022) | NCT03951753 | Tirzepatide (15 mg) | Initiated at 2.5 mg weekly; increased to 5 mg, 7.5 mg, 10 mg, and 12.5 mg every 4 weeks; maintained at 15 mg for 8 weeks | Once weekly | 28 weeks |
| Heise T et al. (2022) | NCT03951753 | Semaglutide (1 mg) | Initiated at 0.25 mg weekly; increased to 0.5 mg weekly for 4 weeks; maintained at 1 mg for 20 weeks | Once weekly | 28 weeks |
| Heise T et al. (2022) | NCT03951753 | PLB | Volume-matched to Tirzepatide and Semaglutide doses | Once weekly | 28 weeks |
| Jiang H et al. (2022) | NCT04466904 | Mazdutide (3.0 mg) | Dose escalation: 1.0 mg (Weeks 1–4) → 2.0 mg (Weeks 5–8) → 3.0 mg (Weeks 9–12) | Once weekly | 12 weeks |
| Jiang H et al. (2022) | NCT04466904 | Mazdutide (4.5 mg) | Dose escalation: 1.5 mg (Weeks 1–4) → 3.0 mg (Weeks 5–8) → 4.5 mg (Weeks 9–12) | Once weekly | 12 weeks |
| Jiang H et al. (2022) | NCT04466904 | Mazdutide (6.0 mg) | Dose escalation: 2.0 mg (Weeks 1–4) → 4.0 mg (Weeks 5–8) → 6.0 mg (Weeks 9–12) | Once weekly | 12 weeks |
| Jiang H et al. (2022) | NCT04466904 | Dulaglutide (1.5 mg) | Fixed dose: 1.5 mg | Once weekly | 12 weeks |
| Jiang H et al. (2022) | NCT04466904 | PLB | Volume-matched placebo following the same dose-escalation schedules as IBI362 groups | Once weekly | 12 weeks |
| Ludvik B et al. (2021) | NCT03882970 | Tirzepatide: 5 mg | Starting dose: 2.5 mg (4 weeks) → Escalated to 5 mg (Week 5 onward) | Once weekly | 52 weeks |
| Ludvik B et al. (2021) | NCT03882970 | Tirzepatide: 10 mg | Starting dose: 2.5 mg (4 weeks) → Escalated to 5 mg (4 weeks) → 10 mg (Week 9 onward) | Once weekly | 52 weeks |
| Ludvik B et al. (2021) | NCT03882970 | Tirzepatide: 15 mg | Starting dose: 2.5 mg (4 weeks) → Escalated to 5 mg (4 weeks) → 10 mg (4 weeks) → 15 mg (Week 13 onward) | Once weekly | 52 weeks |
| Ludvik B et al. (2021) | NCT03882970 | Insulin Degludec | Initial dose: 10 units/day → Weekly titration to fasting blood glucose target (<90 mg/dL) | Once weekly | 52 weeks |
| Nahra R et al. (2021) | NCT03235050 | Cotadutide 100 µg | 100 µg once daily | Once daily | 54 weeks |

| | | | | | |
|---|---|---|---|---|---|
| Nahra R et al. (2021) | NCT03235050 | Cotadutide 200 µg | 100 µg initial dose → Titrated to 200 µg/day | Once daily | 54 weeks |
| Nahra R et al. (2021) | NCT03235050 | Cotadutide 300 µg | 100 µg initial dose → Titrated to 300 µg/day | Once daily | 54 weeks |
| Nahra R et al. (2021) | NCT03235050 | Liraglutide 1.8 mg | 0.6 mg starting dose, up titrated weekly by 0.6 mg until 1.8 mg/day | Once daily | 14 weeks |
| Nahra R et al. (2021) | NCT03235050 | PLB | Volume- and frequency-matched placebo for Cotadutide dosing | Once daily | 14 weeks |
| Parker VE et al. (2020) | NCT03244800 | Cotadutide (Cohort 1 - 300 µg maintained for 28 days (Days 22–49) | 50 µg/day (Days 1–7) → 100 µg/day (Days 8–14) → 200 µg/day (Days 15–21) → 300 µg/day (Days 22–49) | Once daily | 49 days |
| Parker VE et al. (2020) | NCT03244800 | Cotadutide (Cohort 2 - 300 µg maintained for 7 days Days 43–49) | 50 µg/day (Days 1–14) → 100 µg/day (Days 15–28) → 200 µg/day (Days 29–42) → 300 µg/day (Days 43–49) | Once daily | 49 days |
| Parker VE et al. (2020) | NCT03244800 | PLB (cohort 1) | Volume-matched placebo following the same dose-escalation schedules as Cotadutide group | Once daily | 49 days |
| Parker VER et al. (2022) | NCT03550378 | Cotadutide | 50 µg (Days 1–4) → Escalated to 100 µg (Days 5–11) → 200 µg (Days 12–18) → 300 µg (Days 19–32) | Once daily | 32 days |
| Parker VER et al. (2022) | | PLB | Volume- and frequency-matched placebo injections | Once daily | 32 days |
| Rosenstock J et al. (2021) | NCT03954834 | Tirzepatide (5 mg) | 2.5 mg (Weeks 1–4) → Maintained at 5 mg (Week 5 onward) | Once weekly | 40 weeks |
| Rosenstock J et al. (2021) | NCT03954834 | Tirzepatide (10 mg) | 2.5 mg (Weeks 1–4) → 5 mg (Weeks 5–8) → Maintained at 10 mg (Week 9 onward) | Once weekly | 40 weeks |
| Rosenstock J et al. (2021) | NCT03954834 | Tirzepatide (15 mg) | 2.5 mg (Weeks 1–4) → 5 mg (Weeks 5–8) → 10 mg (Weeks 9–12) → Maintained at 15 mg (Week 13 onward) | Once weekly | 40 weeks |
| Rosenstock J et al. (2021) | NCT03954834 | PLB | Volume-matched to Tirzepatide doses | Once weekly | 40 weeks |
| Rosenstock J et al. (2023) - Insulin Lispro | NCT04537923 | Tirzepatide (5 mg) | 2.5 mg (Weeks 1–4) → Maintained at 5 mg from Week 5 onward | Once weekly | 52 weeks |
| Rosenstock J et al. (2023) - Insulin Lispro | NCT04537923 | Tirzepatide (10 mg) | 2.5 mg (Weeks 1–4) → 5 mg (Weeks 5–8) → Escalated to 10 mg from Week 9 onward | Once weekly | 52 weeks |
| Rosenstock J et al. (2023) - Insulin Lispro | NCT04537923 | Tirzepatide (15 mg) | 2.5 mg (Weeks 1–4) → 5 mg (Weeks 5–8) → 10 mg (Weeks 9–12) → Escalated to 15 mg from Week 13 onward | Once weekly | 52 weeks |

| | | | | | |
|---|---|---|---|---|---|
| Rosenstock J et al. (2023) - Insulin Lispro | NCT04537923 | Insulin Lispro | Initial dose: 4 IU before each meal, adjusted based on fasting/pre-prandial glucose levels | Thrice daily | 52 weeks |
| Rosenstock J et al. (2023) - Dulaglutide | NCT04867785 | Retatrutide (0.5 mg) | 0.5 mg group: Fixed | Once weekly | 36 weeks |
| Rosenstock J et al. (2023) - Dulaglutide | NCT04867785 | Retatrutide (4 mg No Escalation Group) | Fixed dose of 4 mg | Once weekly | 36 weeks |
| Rosenstock J et al. (2023) - Dulaglutide | NCT04867785 | Retatrutide (4 mg Escalation Group) | 2 mg (Weeks 1–4) → 4 mg (Weeks 5–36) | Once weekly | 36 weeks |
| Rosenstock J et al. (2023) - Dulaglutide | NCT04867785 | Retatrutide (8 mg Slow Escalation) | 2 mg (Weeks 1–4) → 4 mg (Weeks 5–8) → 8 mg (Weeks 9–36) | Once weekly | 36 weeks |
| Rosenstock J et al. (2023) - Dulaglutide | NCT04867785 | Retatrutide (8 mg Fast Escalation) | 4 mg (Weeks 1–4) → 8 mg (Weeks 5–36) | Once weekly | 36 weeks |
| Rosenstock J et al. (2023) - Dulaglutide | NCT04867785 | Retatrutide (12 mg Escalation Group) | 2 mg (Weeks 1–4) → 4 mg (Weeks 5–8) → 8 mg (Weeks 9–12) → 12 mg (Weeks 13–36) | Once weekly | 36 weeks |
| Rosenstock J et al. (2023) - Dulaglutide | NCT04867785 | Dulaglutide (1.5 mg) | Fixed dose: 1.5 mg | Once weekly | 36 weeks |
| Rosenstock J et al. (2023) - Dulaglutide | NCT04867785 | PLB | Volume-matched placebo following the same dose-escalation schedules as Retatrutide groups | Once weekly | 36 weeks |
| Schiavon M et al. (2021) | NCT02973321 | SAR425899 (0.12 mg) | Fixed dose: 0.12 mg/day | Once daily | 26 weeks |
| Schiavon M et al. (2021) | NCT02973321 | SAR425899 (0.16 mg) | Fixed dose: 0.16 mg/day | Once daily | 26 weeks |
| Schiavon M et al. (2021) | NCT02973321 | SAR425899 (0.20 mg) | Fixed dose: 0.20 mg/day | Once daily | 26 weeks |
| Schiavon M et al. (2021) | NCT02973321 | Liraglutide (1.80 mg) | Fixed dose: 1.80 mg/day | Once daily | 26 weeks |
| Schiavon M et al. (2021) | NCT02973321 | PLB | Volume- and frequency-matched placebo for each active treatment arm | Once daily | 26 weeks |
| Tillner J et al. (2018) | NCT02411825 | SAR425899 (Group Y) | 0.03 mg (Days 1–7) → 0.06 mg (Days 8–14) → 0.09 mg (Days 15–21) | Once daily | 28 days |
| Tillner J et al. (2018) | NCT02411825 | SAR425899 (Group Z) | 0.06 mg (Days 1–7) → 0.12 mg (Days 8–14) → 0.18 mg (Days 15–21) | Once daily | 28 days |
| Tillner J et al. (2018) | NCT02411825 | PLB | Volume-matched placebo following the same dose-escalation schedules as SAR425899 groups | Once daily | 28 days |
| Urva S et al. (2022) | NCT04143802 | Retatrutide: 1.5 mg (Fixed Dose) | 1.5 mg weekly for 12 weeks | Once weekly | 12 weeks |

| | | | | | |
|---|---|---|---|---|---|
| Urva S et al. (2022) | NCT04143802 | Retatrutide: 3 mg (Fixed Dose) | 3 mg weekly for 12 weeks | Once weekly | 12 weeks |
| Urva S et al. (2022) | NCT04143802 | Retatrutide: 3/6 mg (Gradual Escalation) | 3 mg (Weeks 1–4) → 6 mg (Weeks 5–12) | Once weekly | 12 weeks |
| Urva S et al. (2022) | NCT04143802 | Retatrutide: 3/6/9/12 mg (Extended Escalation) | 3 mg (Weeks 1–2) → 6 mg (Weeks 3–4) → 9 mg (Weeks 5–8) → 12 mg (Weeks 9–12) | Once weekly | 12 weeks |
| Urva S et al. (2022) | NCT04143802 | Dulaglutide (1.5 mg) | Fixed dose: 1.5 mg | Once weekly | 12 weeks |
| Urva S et al. (2022) | NCT04143802 | PLB | Volume-matched placebo following the same dose-escalation schedules as Retatrutide groups | Once weekly | 12 weeks |
| Zhang B et al. (2024) | NA | Mazdutide 3 mg | 1.5 mg (Weeks 1–4) → 3 mg (Weeks 5–20) | Once weekly | 20 weeks |
| Zhang B et al. (2024) | NA | Mazdutide 4.5 mg | 1.5 mg (Weeks 1–4) → 3 mg (Weeks 5–8) → 4.5 mg (Weeks 9–20) | Once weekly | 20 weeks |
| Zhang B et al. (2024) | NA | Mazdutide 6 mg | 2 mg (Weeks 1–4) → 4 mg (Weeks 5–8) → 6 mg (Weeks 9–20) | Once weekly | 20 weeks |
| Zhang B et al. (2024) | NA | Dulaglutide (1.5 mg) | Fixed dose: 1.5 mg | Once weekly | 20 weeks |
| Zhang B et al. (2024) | NA | PLB | Volume-matched placebo mimicking the Mazdutide dose-escalation schedules | Once weekly | 20 weeks |
