# Supplementary material for: Beyond GLP-1: efficacy and safety of dual and triple incretin agonists in personalized type 2 diabetes care—a systematic review and network meta-analysis
Source: Acta Diabetol. 2025 Jun 5;62(9):1359–70. doi: 10.1007/s00592-025-02534-y (PMC12433336; doi:10.1007/s00592-025-02534-y)
Supplement: Supplementary file 18 — Attachment 1 Quality Assessment of Included Studies Using the NIH Quality Assessment Tool. Supplementary file18 (DOCX 21 KB) [file 592_2025_2534_MOESM18_ESM.docx]

**Attachment 1** Quality assessment

| Study | Q1 | Q2 | 3 | 4 | 5 | 6 | 7 | 8 | 9 | 10 | 11 | 12 | 13 | 14 | Overall Rating |
| --- | --- | --- | --- | --- | --- | --- | --- | --- | --- | --- | --- | --- | --- | --- | --- |
| Ambery et al., 2018 - GLP-1/Glucagon Dual Agonist Study | Yes | Yes | Yes | Yes | Yes | Yes | Yes | Yes | Yes | Yes | Yes | Yes | Yes | Yes | Good |
| Rosenstock et al., 2023 - Tirzepatide vs. Insulin Lispro in T2DM (SURPASS-6 Trial) | Yes | Yes | No | No | No | Yes | Yes | Yes | Yes | Yes | Yes | Yes | Yes | Yes | Good |
| Dahl et al., 2022 - Tirzepatide vs. Placebo in T2DM (SURPASS-5 Trial) | Yes | Yes | Yes | Yes | Yes | Yes | Yes | Yes | Yes | Yes | Yes | Yes | Yes | Yes | Good |
| Rosenstock et al., 2021 - Tirzepatide Monotherapy vs. Placebo in T2DM (SURPASS-1 Trial) | Yes | Yes | Yes | Yes | Yes | Yes | Yes | Yes | Yes | Yes | Yes | Yes | Yes | Yes | Good |
| Heise et al., 2022 - Tirzepatide vs. Placebo or Semaglutide in T2DM (Phase 1 Trial) | Yes | Yes | Yes | Yes | Yes | Yes | Yes | Yes | Yes | Yes | Yes | Yes | Yes | Yes | Good |
| Frías et al., 2021 - Tirzepatide vs. Semaglutide in T2DM (SURPASS-2 Trial) | Yes | Yes | No | No | No | No | Yes | Yes | Yes | Yes | Yes | Yes | Yes | Yes | Good |
| Del Prato et al., 2021 - Tirzepatide vs. Insulin Glargine in T2DM (SURPASS-4 Trial) | Yes | Yes | No | No | No | Yes | Yes | Yes | Yes | Yes | Yes | Yes | Yes | Yes | Good |
| Garvey et al., 2023 - Tirzepatide for Obesity in T2DM (SURMOUNT-2 Trial) | Yes | Yes | Yes | Yes | Yes | Yes | Yes | Yes | Yes | Yes | Yes | Yes | Yes | Yes | Good |
| Golubic et al., 2023 - Dual GLP-1/Glucagon Receptor Agonist in T2DM and Obesity | Yes | Yes | Yes | Yes | Yes | Yes | Yes | Yes | Yes | Yes | Yes | Yes | Yes | Yes | Good |
| Blüher et al., 2023 - Survodutide vs. Placebo or Semaglutide in T2DM (Phase II Trial) | Yes | Yes | Yes | Yes | Yes | Yes | Yes | Yes | Yes | Yes | Yes | Yes | Yes | Yes | Good |
| Frías et al., 2020 - Tirzepatide Dose-Escalation Regimens in T2DM (Phase 2 Study) | Yes | Yes | Yes | Yes | Yes | Yes | Yes | Yes | Yes | Yes | Yes | Yes | Yes | Yes | Good |
| Jiang et al., 2022 - GLP-1/Glucagon Dual Agonist IBI362 in T2DM (Phase 1b Trial) | Yes | Yes | Yes | Yes | Yes | Yes | Yes | Yes | Yes | Yes | Yes | Yes | Yes | Yes | Good |
| Parker et al., 2020 - Efficacy and Safety of Cotadutide in T2DM (Phase 2a Study) | Yes | Yes | Yes | Yes | Yes | Yes | Yes | Yes | Yes | Yes | Yes | Yes | Yes | Yes | Good |
| Zhang et al., 2024 - Mazdutide in T2DM (Phase 2 Trial) | Yes | Yes | Yes | Yes | Yes | Yes | Yes | Yes | Yes | Yes | Yes | Yes | Yes | Yes | Good |
| Rosenstock et al., 2023 - Retatrutide in T2DM (Phase 2 Trial) | Yes | Yes | Yes | Yes | Yes | Yes | Yes | Yes | Yes | Yes | Yes | Yes | Yes | Yes | Good |
| Urva et al., 2022 - LY3437943 Triple Agonist in T2DM (Phase 1b Trial) | Yes | Yes | Yes | Yes | Yes | Yes | Yes | Yes | Yes | Yes | Yes | Yes | Yes | Yes | Good |
| Ludvik et al., 2021 - Tirzepatide vs. Insulin Degludec in T2DM (SURPASS-3 Trial) | Yes | Yes | No | No | No | Yes | Yes | Yes | Yes | Yes | Yes | Yes | Yes | Yes | Good |
| Asano et al., 2022 - Cotadutide in Japanese T2DM Patients (Phase 1 Study) | Yes | Yes | Yes | Yes | Yes | Yes | Yes | Yes | Yes | Yes | Yes | No | Yes | Yes | Good |
| Tillner et al., 2018 - SAR425899 Dual GLP-1/Glucagon Receptor Agonist in T2DM (Phase 1 Trial) | Yes | Yes | Yes | Yes | Yes | Yes | Yes | Yes | Yes | Yes | Yes | No | Yes | Yes | Good |
| Frias et al., 2018 - LY3298176 (Dual GIP/GLP-1 Agonist) in T2DM (Phase 2 Trial) | Yes | Yes | Yes | Yes | Yes | Yes | Yes | Yes | Yes | Yes | Yes | Yes | Yes | Yes | Good |
| Feng et al., 2023 - Tirzepatide in Chinese Patients with T2DM (Phase 1 Trial) | Yes | Yes | Yes | Yes | Yes | Yes | Yes | Yes | Yes | Yes | Yes | No | Yes | Yes | Good |
| Parker et al., 2022 - Cotadutide in T2DM with CKD (Phase 2a Trial) | Yes | Yes | Yes | Yes | Yes | Yes | Yes | Yes | Yes | Yes | Yes | Yes | Yes | Yes | Good |
| Schiavon et al., 2021 - SAR425899 vs. Liraglutide in T2DM (Phase 2b Study) | Yes | Yes | Yes | Yes | Yes | Yes | Yes | Yes | Yes | Yes | Yes | No | Yes | Yes | Good |
| Furihata et al., 2021 - Tirzepatide in Japanese Patients with T2DM (Phase 1 Trial) | Yes | Yes | Yes | Yes | Yes | Yes | Yes | Yes | Yes | Yes | Yes | No | Yes | Yes | Good |
| Nahra et al., 2021 - Cotadutide in T2DM with Obesity (Phase 2b Study) | Yes | Yes | Yes | Yes | Yes | Yes | Yes | Yes | Yes | Yes | Yes | Yes | Yes | Yes | Good |
| Schmitt et al., 2017 - RG7697 Dual GIP/GLP-1 Agonist in T2DM (Phase 1 Trial) | Yes | Yes | Yes | Yes | Yes | Yes | Yes | Yes | Yes | Yes | Yes | No | Yes | Yes | Good |
